# Promoting Responsible DeepSeek Deployment in Health Care: Scoping Review Comparing Grey and White Literature

**DOI:** 10.2196/80770

**Published:** 2025-12-05

**Authors:** Wang Jiang, Dan Wang, Yihang Zeng, Jiaqi Huang, Chang Xu, Chenxi Liu

**Affiliations:** 1 School of Management, Hubei University of Chinese Medicine, Wuhan, China; 2 School of Pharmacy, Huazhong University of Science and Technology, Wuhan, China; 3 School of Medicine and Health Management, Huazhong University of Science and Technology, Hangkong Road 13, Qiaokou District, Wuhan, Hubei, 430000, China, 86 15623423595; 4 Intelligent Hospital Research Academy, Peking University Shenzhen Hospital, Shenzhen, China

**Keywords:** DeepSeek, hospital, large language model, responsible use, China

## Abstract

**Background:**

DeepSeek is an open-source large language model (LLM), and it has greatly accelerated LLM adoption in health care. Its rapid deployment has sparked concerns regarding its impact on patient outcomes and safety. However, little is known about how DeepSeek is used and regulated in health care.

**Objective:**

This study aimed to (1) systematically review the characteristics of DeepSeek deployed in the top 100 hospitals in China, and (2) compare the performance and risks of DeepSeek between hospital disclosures and research evidence.

**Methods:**

We searched the official websites and WeChat accounts of the top 100 hospitals in China and the databases of Web of Science and PubMed, using the terms “DeepSeek” and “large language models.” Searches were limited to records after January 15, 2025, when DeepSeek was first released. All searches were conducted on May 20, 2025, with an update on June 28, 2025. We extracted the basic characteristics of DeepSeek; its aims, evaluation approach, performance, and risks; and hospital regulations. A coding framework was developed covering the application scenarios, evaluation dimensions, and risk sources of LLMs. The risk of bias was assessed using the Joanna Briggs Institute checklist.

**Results:**

We identified a total of 58 DeepSeek models in 48 out of the top 100 Chinese hospitals and found 27 studies in the literature. The first hospital deployment of DeepSeek was recorded on February 10, 2025, and deployment rapidly expanded to 37 hospitals within a month. Concurrently, most related research studies (20/27, 74%) were published after May 2025. Among deployments and studies that reported version information, DeepSeek-reasoner (R1) was the most frequently used model, and private deployment was the predominant approach. DeepSeek was mainly used to assist in clinical decision-making, including patient diagnosis and treatment recommendation. Among hospital disclosures, only 36% (21/58) clearly indicated a predeployment assessment, 22% (13/58) presented assessment results, and 9% (5/58) identified potential risks and countermeasures. We found poor transparency in hospital reporting, with none of the disclosures presenting evaluation details. Hospitals were more likely to report higher performance and fewer risks for DeepSeek.

**Conclusions:**

This is one of the first scoping reviews to reveal the rapid, widespread deployment of DeepSeek in China’s leading hospitals, primarily for clinical decision support. The deployment of DeepSeek in China’s leading hospitals poses potential risks to patient outcomes and safety. We highlight the urgent need for existing regulations to be expanded to downstream developers and users to promote the responsible use of LLMs in health care. Hospitals need to use a more rigorous validation process and adopt a more transparent reporting policy. The main limitations of this review include the restriction to top-tier hospitals and the inherent constraints of gray literature. These factors should be considered when interpreting the findings.

## Introduction

Large language models (LLMs), such as ChatGPT, are increasingly becoming a transformative force in shaping health care services. As of February 19, 2024, over 500 studies had tested the performance of LLMs across health care applications [1,2], including doctor diagnosis assistance, treatment recommendation, clinical documentation, patient triage and education, and medical research support. With the increase in high-quality datasets and downstream fine-tuning, LLMs have become increasingly comparable with experts in some specific clinical tasks. For example, the Articulate Medical Intelligence Explorer, a conversational diagnostic LLM, demonstrated greater diagnostic accuracy and superior performance compared with primary care physicians in 159 standardized patient cases covering the cardiovascular, respiratory, gastroenterology, neurology, urology, obstetrics and gynecology, and internal medicine fields [3].

Despite rapid advancements in research, the real-world deployment and usage of LLMs in health care remained limited prior to 2025. On one hand, the performance of LLMs in real-world settings has been inconclusive [4]. Bedi et al [2] reviewed 519 evaluation studies of LLMs in health care and found that only 5% of studies used real patient data for validation. On the other hand, LLMs’ hallucinations, namely, fabricating plausible but factually incorrect content, can endanger patient safety [5]. This risk was often overlooked in existing studies. The review by Huo et al [6], which was published in 2025, found that 32.1% (44/137) of studies failed to consider patient safety when LLMs were used as a patient chatbot health adviser. Finally, the restricted permission regarding intellectual property protection, the prerequisite of infrastructure, and the high cost of deployment and usage hinder the real-world application of LLMs [7].

On January 15, 2025, a Chinese artificial intelligence (AI) company released a low-cost and open-source LLM, DeepSeek, which significantly reduces barriers to LLM accessibility. By permitting free commercial use and secondary development, DeepSeek substantially accelerates the wide deployment and application of LLMs in health care. It was reported that over 90 hospitals in China had engaged DeepSeek in health care services by February 25, 2025 [8], only 40 days after its first release. Unlike other proprietary models, DeepSeek has the distinct advantages of being low cost and open source [9]. Evidence suggests that its performance in specific medical tasks is comparable to that of established, proprietary models, highlighting its considerable potential for medical applications [10,11].

However, DeepSeek’s rapid deployment in Chinese hospitals has sparked concerns regarding its impact on patient outcomes and safety [12]. Chen and Miao [8] presented a narrative description of the application of DeepSeek in 90 Chinese hospitals, and Zeng et al [12] qualitatively discussed the urgent need for the validation of DeepSeek in real-world clinical settings and called for a framework to govern trustworthy use of LLMs. However, little is known about the characteristics of DeepSeek deployed in Chinese hospitals, including its application specialty, scenarios, performance, and risks, and the potential corresponding regulations. DeepSeek in Chinese hospitals may aim to support superficial tasks (eg, draft clinical notes), like a few health systems in the United States [13], rather than inform the medical decision-making of physicians (eg, diagnosis and treatment recommendation). Such kind of LLM application is less likely to impact patient outcomes and may alleviate the public concern of its impact on the quality of health care.

While global initiatives, such as the World Health Organization’s ethics and governance guidelines, the US AI Executive Order, and the EU AI Act, provide a foundational regulatory framework [14,15], a rigorous evaluation of DeepSeek’s deployment in health care is critical for guiding its clinical integration and identifying potential risks [9]. This gap currently presents a universal challenge to ensuring responsible AI deployment in health care globally. A direct comparison between real-world deployment data and published research evidence is essential for enhancing the transparency and reliability of the deployment of LLMs, thereby facilitating their more responsible use, which is absent in existing literature [2,16,17]. Although research evidence regarding DeepSeek’s performance in health care has accumulated [18], no study has summarized such evidence and compared it with the performance of DeepSeek deployed in real-world settings.

Therefore, our study aimed to address this research gap. We selected the top 100 Chinese hospitals and compared the performance of their deployed DeepSeek with the performance reported in research. These hospitals were selected because of their well-established public disclosure platforms (eg, official websites and WeChat accounts) and historical early adoption of advanced technologies. Specifically, this study aimed to (1) summarize the characteristics of deployed DeepSeek and assess its performance, risks, and regulations in the top 100 hospitals in China; (2) review the research evidence on the performance of DeepSeek in health care; and (3) compare the results between hospital disclosure and research evidence to inform the responsible deployment and usage of LLMs in real-world health care settings.

## Methods

### Study Design

We performed a scoping review based on both gray and white literature. We first searched hospital disclosures related to DeepSeek deployment from Chinese hospitals’ official websites and WeChat accounts. We simultaneously identified studies assessing DeepSeek performance and risks in health care from literature datasets. Based on the retrieved information, we summarized the characteristics, performance, and risks of hospital-deployed DeepSeek, and compared them with the results from existing studies. The PRISMA-ScR (Preferred Reporting Items for Systematic Reviews and Meta-Analyses extension for Scoping Reviews) checklist is provided in Checklist 1.

We restricted our search to the top 100 hospitals based on China’s hospital rankings (2023 version) [19]. The list serves as an important reference to assess the overall clinical and research quality of Chinese hospitals, as it ranks hospitals based on clinical experts’ evaluations, as well as the hospitals’ research inputs and outputs.

### Search Strategy and Eligibility Criteria

We first searched the official websites and WeChat accounts of the top 100 hospitals in China. The search terms were “DeepSeek,” “artificial intelligence/AI,” and “large language models” in Chinese. For each hospital, we systematically searched the official website and official WeChat account (verified based on official QR codes or redirect links published on the respective hospital website) to obtain publicly disclosed information regarding DeepSeek deployment. To ensure traceability, if both the official website and official WeChat account disclosed information about the same model, the source with the most comprehensive information was prioritized as the primary entry. If a hospital published multiple articles regarding the same deployment model, the “first official disclosure” was defined as the deployment date. If subsequent posts provided additional key information (eg, version, application scenarios, and metrics), such information was merged into the same model. All source links and the corresponding hospital list are provided in Table S2 in Multimedia Appendix 1.

We then performed a supplementary search in Web of Science and PubMed to identify research studies related to DeepSeek. The search terms included “DeepSeek/large language models,” “evaluation/assessment,” and “health care/hospitals” (Table S1 in Multimedia Appendix 1). Since DeepSeek was first released on January 15, 2025, we restricted all searches to articles published after this date. All searches were first performed on May 20, 2025, and updated searches were performed on June 28, 2025.

Regarding disclosures from hospitals’ official websites and WeChat accounts, we included websites meeting the following criteria: (1) clearly reported DeepSeek deployment in the hospital, and (2) included information regarding DeepSeek’s application specialty, scenario, performance, risk, or management. We excluded websites that (1) presented a general description of DeepSeek, (2) indicated DeepSeek was intended to be used, and (3) provided training and education information on DeepSeek.

Among the retrieved studies, we included those that (1) aimed to assess DeepSeek’s performance or risks, (2) used DeepSeek in health care settings, and (3) evaluated DeepSeek’s performance based on clinical cases rather than medical exams (eg, United States Medical Licensing Examination). We excluded studies that (1) focused on DeepSeek performance in nonmedical scenarios, (2) assessed other LLMs rather than DeepSeek, and (3) did not present original results of DeepSeek’s performance.

### Data Extraction and Coding

Based on data from hospital disclosures and research studies, we extracted the following information: basic characteristics of the hospital (eg, name and province) and DeepSeek (eg, version and deployment date), aims (eg, application specialty and scenarios), performance evaluation (eg, evaluation approach and metrics), risks (eg, hallucination), and regulations (eg, performance monitoring).

Based on reviews of LLMs in health care by Bedi et al [2] and Zeng et al [12], we developed a coding system for categorizing DeepSeek’s application scenarios, evaluation dimensions, and risks. All collected information was mapped into a structural data sheet based on the coding system. Specifically, DeepSeek’s application scenarios were classified into the following 3 categories with 12 subdomains: assisting medical decision-making (diagnosis formulation, treatment and medication recommendation, clinical documentation, and hospital administrative documentation), patient management (patient education, adherence supporting, health monitoring, and appointment coordination), and medical research and education (literature review, knowledge assessment, medical education, and protocol assistance).

The evaluation dimensions of DeepSeek’s performance were classified into accuracy (how close DeepSeek’s response is to the true or expected response), comprehensiveness (how well DeepSeek’s response addresses all aspects of the task), factuality (whether DeepSeek’s response originates from a verifiable and citable source), robustness (DeepSeek’s resilience to perturbations, eg, typos), fairness (how well DeepSeek’s output is equitable across diverse populations), deployment metrics (technical evaluation of DeepSeek to generate a response, eg, response time), and uncertainty (how uncertain DeepSeek is about its output) [2].

The risks of DeepSeek use were classified into the following aspects: hallucination, inappropriate recommendation, clinician overreliance, inconsistent quality of responses, misalignment regarding patient needs and conditions, miscommunication of benefits or risks, outdated knowledge reinforcement, and fabricated citations. During the coding process, if the coding system did not cover the information, a new code was created.

All dimensions were scored using a binary scheme (yes/no or not mentioned) based on explicit disclosures. The details of the coding framework and its interpretation are presented in Multimedia Appendix 1.

We used a dual-coding approach to ensure data integrity. One coder (WJ) performed all initial coding, which was subsequently subjected to an independent, full verification by a second coder (CL). Inconsistency was resolved by discussion and consensus. This process yielded 23 initial discrepancies from a total of 348 coding decisions, corresponding to a 6.6% discrepancy rate.

### Analysis

We have presented descriptive results based on number (%) and median (IQR) for categorical and continuous variables, respectively. Our analytical approach involved a narrative comparison to contrast the portrayal of DeepSeek in hospital disclosures versus academic studies. The comparison encompassed the following three primary domains: (1) deployment characteristics (type, version, and department), (2) performance metrics (accuracy, comprehensiveness, factuality, robustness, fairness, and deployment metrics), and (3) documented risks. In addition, the accuracy rate of DeepSeek was compared between hospital disclosures and academic studies. As studies adopted various scales to assess DeepSeek’s performance, to ensure the comparability of results, we adopted a minimum-maximum normalization approach to convert raw results into a range of 0 to 1. For example, assuming DeepSeek’s accuracy performance was 4.5 according to clinical experts based on a 5-point Likert scale (minimum: 1, maximum: 5), the standardized performance would be calculated as follows: ([4.5 − 1.0] / [5.0 − 1.0]) × 100 = 87.5%. All analyses were performed using STATA version 14.0 (StataCorp).

### Ethical Considerations

This study is exempt from ethics approval because it exclusively relies on publicly available information that is legally accessible to the public and protected by law, contains no identifiable personal data, and does not have any privacy issues.

## Results

### Characteristics of Hospital-Deployed DeepSeek and Evidence From Studies

**Figure 1.**
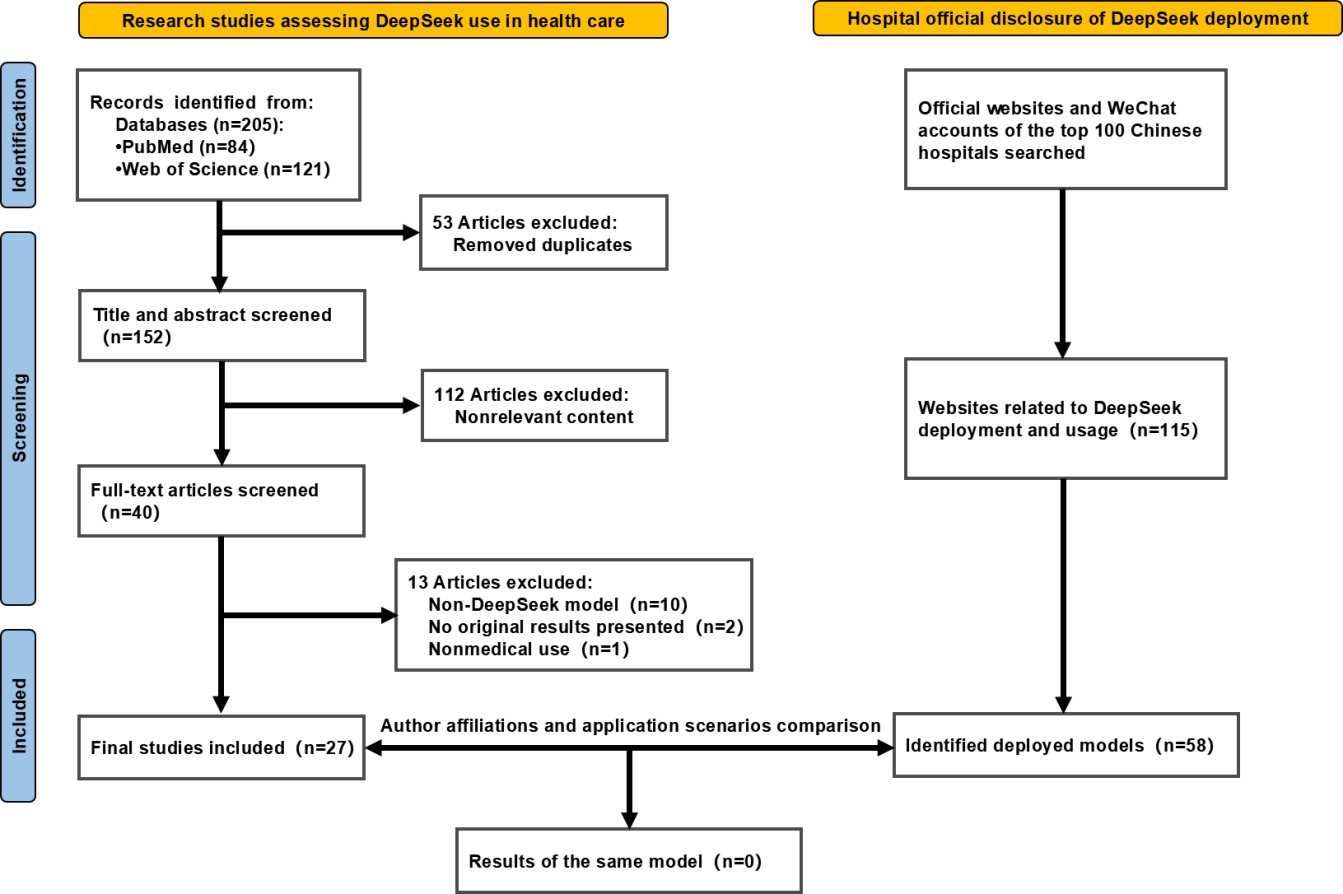
PRISMA (Preferred Reporting Items for Systematic Reviews and Meta-Analyses) flow diagram of the identification process of DeepSeek deployment disclosures and research studies. The scoping review included 58 hospital deployment disclosures and 27 research studies.

By searching hospital official websites and WeChat accounts, 115 websites related to DeepSeek were retrieved from the top 100 hospitals in China (Table S2 in Multimedia Appendix 1). There were 58 DeepSeek models deployed in 48 hospitals from 18 provinces. A total of 205 studies were retrieved from literature datasets, and 27 studies were finally included. We identified 2 studies in which 1 of the authors was from the top 100 hospitals in China (Figure 1). A quality appraisal of the white literature was conducted (Figure S1 in Multimedia Appendix 1). However, none of the studies assessed hospital-deployed DeepSeek models due to different intended application specialties and scenarios.

**Figure 2.**
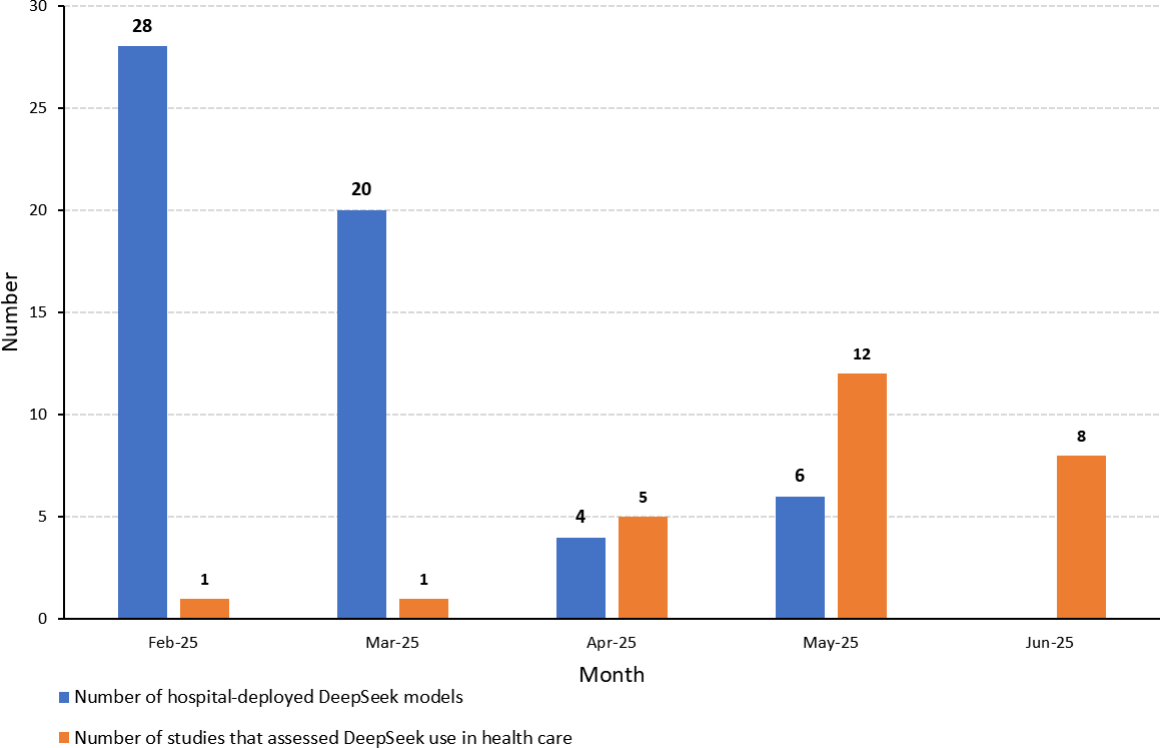
Comparative trends of the numbers of hospital-deployed DeepSeek models and published studies that assessed DeepSeek use in health care from February 2025 to June 2025.

The first DeepSeek deployment in a Chinese hospital was at the Third Affiliated Hospital of Sun Yat-Sen University on February 10, 2025, and the number of deployments rapidly increased to 37 (64%) in the following month (before March 10, 2025), covering 33 hospitals from 16 provinces (Table S3 in Multimedia Appendix 1). The first study regarding the evaluation of DeepSeek’s performance in health care was published on February 28, 2025 [20]. Only 2 studies were published before April 2025 [20,21]. Most studies were published after May 2025 (20/27, 74%; see Table S4 in Multimedia Appendix 1). The comparative trends of the numbers of hospitals deploying DeepSeek models and studies assessing DeepSeek use in health care are presented in Figure 2.

Most hospital deployments (32/58, 55%) did not disclose the version of the deployed DeepSeek, and the rest (26/58, 45%) reported applying DeepSeek-reasoner (R1). Most hospital deployments (37/58, 64%) involved private deployment, and 1 hospital synchronously adopted cloud-based deployment. The remaining hospital deployments (21/58, 36%) did not disclose such information. Among the 27 studies, 16 (59%) assessed DeepSeek-reasoner (R1), 11 (41%) assessed DeepSeek-chat (V3), and 3 (11%) did not mention the DeepSeek version (Table 1).

**Table 1.** Characteristics, performance, and risks of DeepSeek in hospital disclosures and published studies.

Variable	Hospital disclosures (n=58), n (%)	Published studies (n=27), n (%)
DeepSeek version		
DeepSeek-reasoner (R1)	26 (45)	16 (59)
DeepSeek-chat (V3)	0 (0)	11 (41)
Not specified	32 (55)	3 (11)
Deployment type		
Private^a^	37 (64)	—^b^
Not specified	21 (36)	—
Department		
General	41 (71)	4 (15)
Oncology	4 (7)	1 (4)
Pediatrics	2 (3)	1 (4)
Respiratory and critical care medicine	2 (3)	0 (0)
Urology	2 (3)	0 (0)
Rare diseases	2 (3)	0 (0)
Surgery	1 (2)	9 (33)
Gastroenterology	1 (2)	2 (7)
Ophthalmology	0 (0)	2 (7)
Others^c^	2 (3)	7 (26)
Application scenarios		
Clinical decision support		
Diagnosis formulation	35 (60)	8 (30)
Treatment and medication recommendation	23 (40)	7 (26)
Clinical documentation	29 (50)	0 (0)
Hospital administration support	13 (22)	0 (0)
Patient management and service		
Patient education	6 (10)	12 (44)
Treatment adherence support	8 (14)	0 (0)
Health monitoring	1 (2)	1 (4)
Appointment coordination	28 (48)	0 (0)
Research and teaching		
Literature synthesis	5 (9)	1 (4)
Training medical trainee	3 (5)	6 (22)
Research protocol support	4 (7)	0 (0)
Clinical knowledge assessment	0 (0)	0 (0)
Evaluation dimension		
Accuracy	12 (21)	27 (100)
Comprehensiveness	0 (0)	11 (41)
Fairness	0 (0)	6 (22)
Factuality	0 (0)	1 (4)
Deployment metrics	1 (2)	0 (0)
Potential risks		
Hallucination	5 (9)	2 (7)
Inappropriate recommendation	1 (2)	13 (48)
Misaligned recommendations regarding patient needs and conditions	1 (2)	5 (19)
Fake citation	0 (0)	2 (7)
Sensitive to prompts	0 (0)	1 (4)
Outdated knowledge	1 (2)	1 (4)
Poor performance	0 (0)	1 (4)

a One hospital had private and cloud-based deployments.

b Not applicable.

c Others: cardiology, anesthesiology, traditional Chinese medicine, dentistry, hepatology, multidisciplinary clinic, radiology, rehabilitation, and otolaryngology.

### Application Scenarios

Most hospital deployments (41/58, 71%; Table 1) did not report an intended use specialty for DeepSeek, while the rest specified usage in oncology (4/58, 7%), pediatrics (2/58, 3%), respiratory and critical care medicine (2/58, 3%), urology (2/58, 3%), etc. In contrast, existing studies mainly assessed DeepSeek’s usage in surgery (9/27, 33%) and evaluated general use without specialty restriction (4/27, 15%; Table 1).

In terms of application scenarios (Table 1), hospital-deployed DeepSeek was mainly intended to assist with diagnosis formulation (35/58, 60%), support clinical documentation (29/58, 50%), coordinate patient appointments (28/58, 48%), and recommend treatment and medication (23/58, 40%). On the other hand, existing studies mainly used DeepSeek to educate patients (12/27, 44%), assist with diagnosis formulation (8/27, 30%), recommend treatment and medication (7/27, 26%), and train medical trainees (6/27, 22%).

### Performance

Among the 58 deployed models from hospital disclosure, 21 (36%) reported results of predeployment assessment. However, none of them presented evaluation approaches, validation datasets, and metric calculation methods. Most studies assessed DeepSeek’s performance based on simulated cases (22/27, 82%) or real-world cases (5/27, 19%). The median sample size of validation datasets was 21 (IQR 10‐58). Existing studies mostly used human evaluation (by experts and researchers; 22/27, 82%), followed by ground truth comparison (8/27, 30%) and text assessment (5/27, 19%; Table S6 in Multimedia Appendix 1).

### Hospital Disclosure Performance

Among the 58 deployed models, 13 (22%) reported the results of predeployment assessment (Table S5 in Multimedia Appendix 1). Accuracy was the most frequently reported performance metric across both hospital disclosures (12/58, 21%) and studies (11/27, 41%) [8,18,22-30]. The evaluation indicators included accuracy (12/58, 21%) and deployment metrics (1/58, 2%) for hospital disclosure performance.

**Figure 3.**
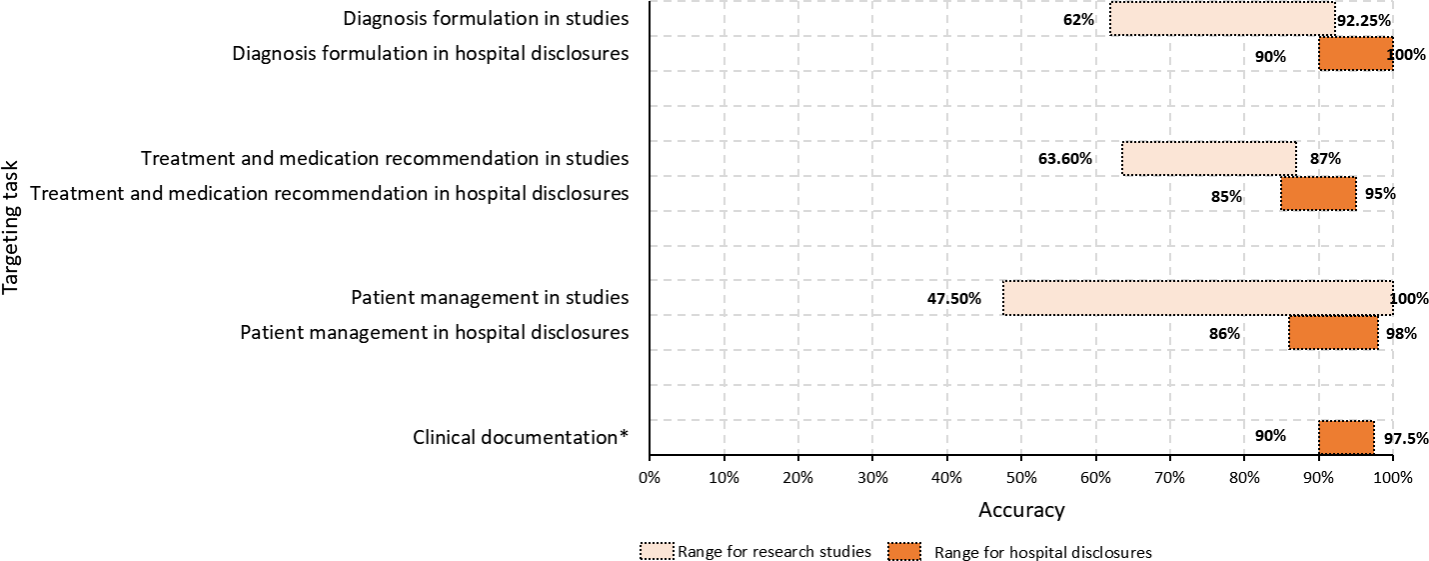
Comparative accuracy of DeepSeek in hospital deployment disclosures versus research studies across 4 targeting tasks: diagnosis formulation, treatment and medication recommendation, patient management, and clinical documentation (scale of 0%‐100%). To standardize results for comparison, scores are converted to percentages through linear transformation in research studies. The conversion formula is as follows: percentage = ([score − minimum score] / [maximum score − minimum score]) × 100. *A comparison is not included for clinical documentation as research studies lacked accuracy data for clinical documentation.

Hospital disclosures often highlighted DeepSeek’s performance in specific clinical applications. Specifically, DeepSeek’s reported accuracy ranged from 90% (myasthenia gravis and cardiac amyloidosis) to 100% (negative pathological section) in diagnosis formulation, 85% (general use without restriction of a specialty) to 95% (pulmonary diseases) in treatment and medication recommendation, 90% (patient information classification) to 97.5% (summary of patient follow-up) in clinical documentation, and 86% (patient self-triage) to 98% (patient education) in patient management. One hospital reported that private DeepSeek deployment reduced response time (<3 s; deployment metrics) (Table S5 in Multimedia Appendix 1 and Figure 3).

### Research-Reported Performance

In terms of studies, the assessment extended beyond accuracy to include comprehensiveness (11/27, 41%) [8,18,22-30], fairness (6/27, 22%) [22,23,26,30-32], and factuality (1/27, 4%) [33]. For diagnostic tasks, when compared to a ground truth, studies have reported accuracy ranging from 62.0% to 88.9% [29,34-37]. On the other hand, these figures ranged from 75.5% to 92.5% when evaluated by clinical experts [18,29,33,38]. In terms of treatment and medication recommendation, studies reported that DeepSeek’s accuracy ranged from 63.9% to 87.0% when considering the best performance evaluated by clinical experts (Table S6 in Multimedia Appendix 1) [18,20,21,25,26,37,38]. When DeepSeek was used to educate patients and medical trainees, studies demonstrated that its response accuracy ranged from 47.5% to 100.0% [8,22-24,27,28,30-32,39-41].

Ali [39] showed that 4.0% of DeepSeek’s responses contained factually incorrect information when used for education regarding lacrimal drainage disorders. DeepSeek’s equity was also evaluated based on differences in responses across genders and ages or text readability. Gurbuz et al [31] showed no significant difference in DeepSeek’s generated educational information for total knee arthroplasty across patient gender and age, and another 4 studies indicated that DeepSeek’s responses generally required a middle school education level (8/9 grade based on the Flesch-Kincaid Grade Level) among patients (Table S6 in Multimedia Appendix 1) [22,23,30,32].

Two studies assessed DeepSeek’s performance in literature synthesis and clinical knowledge assessment (Table S6 in Multimedia Appendix 1) [42,43]. DeepSeek showed satisfactory performance in research screening for meta-analysis (precision: 72.1%‐100%; recall: 58.4%‐94.6%; *F*_1_-score: 58.4%‐87.6%) [42]. However, DeepSeek showed suboptimal performance in another study focusing on medical ontology mapping to create and maintain medical knowledge graphs (precision: 25.8%; recall: 32.7%; *F*_1_-score: 28.8%) [43].

### Risk and Regulation

Regarding hospital disclosures, the mentioned DeepSeek risks included hallucination (5/58, 9%), inappropriate recommendation (1/58, 2%), outdated knowledge reinforcement (1/58, 2%), and misaligned content for patient needs and conditions (1/58, 2%). In contrast, the most mentioned risk in studies was inappropriate recommendation (13/27, 48%) [18,20,25-28,30-32,34,37-39], following by misaligned content for patient needs and conditions (5/27, 19%) [25,26,30-32], hallucination (2/27, 7%) [38,41], fake citation (2/27, 7%) [33,41], outdated knowledge reinforcement (1/27, 4%) [28], and sensitivity to prompt change (1/27, 4%) [36] (Table 1).

We noted that Fudan University Children’s Hospital had established an evaluation framework and used it to monitor DeepSeek use in supporting clinical decision-making. However, no specific details were presented. Five hospitals reported that they had implemented 1-4 risk management measures, including selection of training data (5/48, 10%), data update and training (2/48, 4%), citation tracing (1/48, 2%), chain-of-thought fine-tuning (1/48, 2%), mandating physician’s final accountability (1/48, 2%), and setting standardized procedures for the use of DeepSeek with patients (1/48, 2%).

## Discussion

### Principal Findings

To the best of our knowledge, this is the first study that has systematically reviewed gray and white literature to summarize the characteristics of DeepSeek deployed in China’s hospitals and compared the performance of these models with that reported in existing studies. We observed the prevalence of DeepSeek deployment in China’s top 100 hospitals, and DeepSeek was mainly intended to assist clinical decision-making, such as patient diagnosis and treatment. An accelerating trend is evident in the deployment of LLMs within health care. This is underscored by both the research landscape and real-world adoption. Multiple systematic reviews have confirmed the rapid integration of LLMs like ChatGPT into key areas such as patient care, clinical decision-making, and medical education [4,44,45]. This pattern is reinforced by the swift adoption of LLMs in clinical practice. For example, DeepSeek’s AI model was deployed in nearly 90 prominent tertiary hospitals across more than 20 provincial-level regions within months of its release in China [8].

However, such use occurred under substantial uncertainty of DeepSeek’s performance and risks in health care, with only 36% (21/58) of hospital-deployed models clearly indicating a predeployment assessment, 22% (13/58) presenting assessment results, and 9% (5/58) identifying potential risks and countermeasures. For the data from most hospital-deployed DeepSeek models, there was limited evidence from published studies to confirm efficacy and safety. We also found poor transparency and reporting regarding the predeployment assessment of hospital-deployed DeepSeek models, as none of the disclosures presented details of the evaluation dataset, approach, and calculation of indicators. In contrast, studies adopted a more rigorous evaluation method and a more comprehensive perspective to assess DeepSeek’s performance and risks.

Our results demonstrated that accuracy was the most frequently reported performance metric in both hospital disclosures and studies [2,45]. This focus aligns with the existing literature, where accuracy emerges as the most commonly assessed parameter. However, performance metrics, such as fairness, bias, deployment metrics, and ethical considerations, remain far less frequently studied [2,45]. In contrast to the included studies, we found that hospitals failed to assess DeepSeek’s comprehensiveness, factuality, and equity, and they were likely to report higher performance and fewer risks for DeepSeek. These situations imply potential threats to patient safety and highlight the urgent need for comprehensive regulations to ensure responsible usage of LLMs in health care.

Our results are broadly consistent with previous concerns of the impact of DeepSeek’s rapid usage on clinical safety and efficacy, with Zeng et al [12] highlighting that real-world usage without a complete evaluation of DeepSeek’s performance endangers clinical decisions. We expanded the understanding of this issue by detailing the landscape and characteristics of DeepSeek deployed in the top 100 hospitals in China. We observed that 64% (37/58) of DeepSeek deployments in hospitals did not report a predeployment assessment, and only 9% (5/58) reported on DeepSeek’s potential risks. In contrast, 63% (17/27) of studies highlighted the risks of DeepSeek use in health care, and the most frequently mentioned risk was inappropriate recommendation (n=13), directly contradicting the fact that over half of hospital-deployed DeepSeek models were intended to assist with diagnosis and treatment recommendation. Unlike studies that adopted broader evaluation dimensions, hospitals only considered DeepSeek’s accuracy and neglected the comprehensiveness, factuality, and equity of its responses. We also observed that certain risks, including clinical overreliance and patient miscommunication (eg, overstating and understating) of benefits or risks, were overlooked by studies and hospitals [2,12].

Our findings revealed a trend wherein performance metrics reported in hospital disclosures, particularly for diagnosis and treatment recommendation, often occupied the higher end of the spectrum observed in the included studies. In our sample, hospitals reported accuracy rates of 90% to 100% for diagnosis and 85% to 95% for treatment recommendation. The broader range of accuracy (eg, 65.0%‐100% and 46.9%‐87.0%) noted in studies indicates that evaluations were conducted under more varied and standardized test conditions. This observed trend is likely attributable to fundamental differences in objectives, validation datasets (internal or external), evaluation approaches (human evaluation or ground truth) [46], and the calculation of indicators [47]. Hospital evaluations may use internal, curated datasets and optimization for specific clinical workflows, whereas independent studies often adopt external benchmarks and rigorous, blinded expert evaluations to assess general-world performance. However, due to poor transparency and reporting from hospitals, we were unable to investigate the underlying reasons.

Compared with the included studies, hospitals need to adopt more transparent reporting procedures, more rigorous evaluation methods, and more comprehensive evaluation frameworks in their future predeployment assessments of LLMs to accurately estimate LLMs’ performance and risks for ensuring responsible use in health care. Furthermore, hospitals may underreport evaluation details due to confidentiality agreements or the early stage of deployment. This lack of transparency is a significant issue that amplifies the risks of deploying LLMs in health care. Consequently, our study results may have a certain degree of underestimation due to the possible existence of nonpublic internal evaluations.

This discrepancy can be further attributed to institutional and policy factors within the Chinese health care system. First, the low-cost and open-source nature of DeepSeek has spurred unprecedented adoption in medical institutions. This momentum is amplified by social media, creating competitive pressure to adopt the technology rapidly to avoid being perceived as technologically backward [12]. This may lead to a selective reporting bias, where successes are highlighted and limitations are understated. Furthermore, this situation could worsen with the intense global AI competition. Second, despite widespread optimism about the prospects of clinical AI, a significant knowledge gap remains, likely leading to an underestimation of LLM-specific risks [48]. Furthermore, the current regulatory policy primarily focuses on upstream technology providers [12]. In contrast, it imposes relatively few direct constraints on downstream users in areas such as model deployment, evaluation, and application. The regulatory gap may permit clinical institutions to overstate model capabilities with limited oversight.

### Implications

Despite the absence of a universally accepted framework for responsible AI deployment in health care, a broad range of pillars of responsible AI deployment have been identified based on previous efforts [49], including accountability (responsibility and auditability during and after AI development), diversity, nondiscrimination and fairness (no reproduction of discrimination or unfairness), human agency and oversight, privacy and data governance (managing data access, data quality, and data privacy), transparency, and social well-being (ubiquitous exposure to social AI systems in all areas of society). Compared with our findings, critical gaps were identified in both the disclosure practices of hospitals and the evaluation focus of academic studies in health care. Based on our findings and comparisons with previous work, we have summarized the key gaps and future steps of related stakeholders in responsible AI deployment in health care (Table 2).

**Table 2.** Implications for responsible large language model deployment in health care.

Current status of AI^a^ deployment in clinical settings, domain, and target audience			Key countermeasures
Narrow focus on highly variable accuracy metrics and severe lack of methodological transparency			
Methodological transparency and evaluation			
Hospitals			Move beyond narrow accuracy metrics to a comprehensive assessment, including accuracy, comprehensiveness, factuality, robustness, fairness, deployment metrics, and uncertainty. Disclose detailed evaluation methodologies, covering validation datasets, approaches, and indicator calculations.
Policy makers			Mandate public disclosure of training and validation processes, including dataset characteristics, evaluation procedures, indicators, analysis, performance, risks, and mitigation plans. Require independent third-party verification of all disclosed information.
Largely ignored or not disclosed aspects			
Privacy and data governance			
Hospitals			Adopt and operationalize standardized approaches or frameworks (eg, DEAL^b^ Checklist and FAIR-AI^c^) for implementation and monitoring. Conduct continuous risk monitoring for data breaches or misuse.
Human agency and oversight			
Hospitals			Establish infrastructure for continuous user feedback and error reporting. Implement formal review criteria for AI-assisted decisions.
Policy makers			Extend regulations to mandate oversight mechanisms in downstream clinical settings.
Regulatory framework and compliance			
Policy makers			Bridge the gap between rapid AI development and existing oversight frameworks. Extend regulatory scope to hold downstream developers and end-user hospitals accountable for model validation and outcome reporting.

a AI: artificial intelligence.

b DEAL: Development, Evaluation, and Assessment of Large Language Models.

c FAIR-AI: Framework for the Appropriate Implementation and Review of AI.

Specifically, the current state of DeepSeek deployment is characterized by a narrow focus on highly variable accuracy metrics, and many critical metrics were largely ignored or not disclosed, including transparency, human agency and oversight, privacy and data governance, and social well-being. Hospitals should adopt a comprehensive assessment, including accuracy, comprehensiveness, factuality, robustness, fairness, deployment metrics, and uncertainty.

Privacy and data governance entail the adoption and operationalization of standardized approaches or frameworks, such as the Development, Evaluation, and Assessment of Large Language Models (DEAL) Checklist and the Framework for the Appropriate Implementation and Review of AI (FAIR-AI) [50-52]. These emerging frameworks play a key role in facilitating AI evaluation in medical settings by providing resources, structures, criteria, and template documents. Concurrently, to ensure human agency and oversight, it is imperative to establish infrastructure for continuous user feedback, mechanisms for reporting inaccuracies, and approaches for formal review criteria establishment [53].

Efforts to strengthen regulatory oversight of AI in health care must be accelerated. The rapid development of AI technology is outpacing the existing regulatory oversight and governance framework from China and the global authority [12]. DeepSeek is being increasingly integrated with existing hospital information systems without corresponding regulatory adaptation [12]. While the Chinese government issued the “Provisional Measures for Generative Artificial Intelligence Services Management” (August 15, 2023), which establishes crucial requirements for upstream technique providers, it does not adequately address downstream implementation [54]. To bridge this regulatory gap and ensure safe, effective clinical integration, its scope should be extended to mandate compliance from downstream developers and end-user hospitals, specifically in the domains of model validation, continuous risk monitoring, and transparent outcome reporting. We recommend a mandated and comprehensive public disclosure for LLMs [2], which should encompass their training and validation processes, including dataset characteristics, evaluation procedures, indicators, and analysis, in addition to their performance, risks, and mitigation plans. All disclosed information must undergo an independent third-party assessment to ensure credibility.

### Strengths and Limitations

The strength of our study is the systematic gray and white literature review of the characteristics of hospitals that deployed DeepSeek and the evidence from previous studies. Using information retrieved from multiple sources, we could depict the landscape of DeepSeek deployment and usage in China’s hospitals and identify the gaps between hospital disclosures and research evidence. Another strength is the coding framework based on the findings of Bedi et al [2] and Zeng et al [12], which enabled us to comprehensively understand the characteristics, performance, risks, and potential countermeasures of DeepSeek.

Our study has some limitations. First, hospitals not listed in the top 100 hospital list have deployed DeepSeek [55]. However, considering the availability of hospital official websites and WeChat accounts as well as deployment likelihood, we limited our study to the top 100 hospitals in China. It is expected that these hospitals are the leading force in the adoption and dissemination of new medical technology and have better-established disclosure platforms for their innovations. Thus, we inferred that the current situation of poor evaluation and reporting of DeepSeek’s performance and risks might be worse in other hospitals, and this warrants further study for confirmation. The generalizability of our findings to other health care settings may be limited, and caution should be exercised when applying these results beyond top-tier hospitals. Second, it is possible that hospitals performed a predeployment evaluation but did not disclose the details. However, considering the limited time between DeepSeek’s first release and the date of hospital DeepSeek deployment, we believe that most hospitals deployed DeepSeek without a comprehensive assessment of its performance and risks. Third, it is important to note that DeepSeek’s deployment does not indicate its full use in hospitals, and its impacts on patient outcomes and safety in real-world settings require further investigation. However, the increasing accessibility of LLMs will, to some extent, impact physicians’ decision-making in clinical practice. Thus, comprehensive regulations and risk management of LLM use are becoming increasingly urgent. Additionally, a credibility assessment of the gray literature was currently impossible due to the limited and heterogeneous data available. This limitation should be addressed in future studies as more evidence accumulates. Fourth, our gray literature search may have omitted DeepSeek deployment that was not published in the official information channels of the top 100 hospitals. However, we prioritized verifiable, institutionally released records over unverifiable content from nonofficial platforms. This strategy ensured that all included information pertained to confirmed institutional deployments, thereby strengthening the credibility of our data.

### Conclusions

We noted the rapid deployment of DeepSeek in China’s top 100 hospitals. However, such an application poses potential risks to patient outcomes and safety due to incomprehensive evaluations of DeepSeek’s performance and risks, and poor reporting of validation methods. Compared with the findings in previous studies, hospitals tend to overstate DeepSeek’s performance while underreporting its risks. This situation highlights the urgent need for a comprehensive governance framework to regulate hospitals and ensure the responsible deployment and use of LLMs.

## Supplementary material

10.2196/80770 Multimedia Appendix 1 Additional information to support the findings of the study.

10.2196/80770 Checklist 1 PRISMA-ScR checklist

## Abbreviations

AI: artificial intelligence
LLM: large language model
