# Supplementary material for: Promoting Responsible DeepSeek Deployment in Health Care: Scoping Review Comparing Grey and White Literature
Source: J Med Internet Res. 2025 Dec 5;27:e80770. doi: 10.2196/80770 (PMC12680131; doi:10.2196/80770)
Supplement: Multimedia Appendix 1 [file jmir-v27-e80770-s001.docx]

**Multimedia Appendix 1.** Additional information to support the findings of the study.

**Table S1. Search strategies for studies assessing DeepSeek performance in health care**

| **Dataset** | **Search strategies** | **Results** |
| --- | --- | --- |
| PubMed | ((("deepseek"[Title/Abstract] OR "large language model"[Title/Abstract] OR "chatgpt"[Title/Abstract]) AND ("deploy*"[Title/Abstract] OR "use"[Title/Abstract] OR "application"[Title/Abstract]) AND ("health care"[Title/Abstract] OR "hospital"[Title/Abstract] OR "primary care"[Title/Abstract]) AND ("china"[MeSH Terms] OR "china"[All Fields] OR "china s"[All Fields] OR "chinas"[All Fields] OR ("chinese people"[Supplementary Concept] OR "chinese people"[All Fields] OR "chinese"[All Fields] OR "chineses"[All Fields]))) OR ("deepseek"[Title/Abstract] AND ("assess*"[All Fields] OR "evaluat*"[All Fields]))) AND (2025/1/15:2025/6/28[pdat]) | 84 |
| Web of Science | ((TS=(deepseek) AND (ALL=(assess*)) OR ALL=(evaluat*)) OR (((TS=(large language model)) OR TS=(chatgpt)) OR TS=(deepseek) AND ((TS=(deploy*)) OR TS=(use*)) OR TS=(application) AND ((TS=(health care)) OR TS=(hospital)) OR TS=(primary care) AND (ALL=(China)) OR ALL=(Chinese)) AND DOP=(2025-01-15/2025-06-28)) | 121 |

**Table S2. Information sources regarding deployed DeepSeek in Chinese top 100 hospitals**

| **Hospital deployed models** | **Information sources** |
| --- | --- |
| DeepSeek at The Third Affiliated Hospital of Sun Yat-sen University | 1. https://www.zssy.com.cn/article/27353  2. https://www.zssy.com.cn/article/27398  3. https://m.itouchtv.cn/program/374/10/909294?shareId=Yue2fdBD |
| DeepSeek at Shanghai Sixth People's Hospital | 1. https://www.6thhosp.com/Content/News/9492  2. https://mp.weixin.qq.com/s/EUVShCsYyYIW1uxg0oZfZQ  3. https://mp.weixin.qq.com/s/vM24gxUFNPwvlvdVjttLwA |
| Qilu Clinical Skills Large Model at Qilu Hospital of Shandong University | 1. https://www.qiluhospital.com/show-26-39475-1.html |
| DeepSeek at Nanfang Hospital of Southern Medical University | 1. https://www.nfyy.com/xwzx/yyxw/a_116598.html  2. https://news.smu.edu.cn/info/1012/109476.htm  3. https://news.smu.edu.cn/info/1019/109226.htm  4. https://news.smu.edu.cn/info/1014/109876.htm  5. https://news.smu.edu.cn/info/1019/111166.htm  6. https://news.smu.edu.cn/info/1019/110916.htm  7. https://news.smu.edu.cn/info/1019/111366.htm |
| “Xiehe Taichu” at Peking Union Medical College Hospital | 1. https://www.pumch.cn/detail/40025.html  2. https://baijiahao.baidu.com/s?id=1824496022025685051&wfr=spider&f  or=pc |
| “Xiaohong AI” Patient Assistant at Obstetrics and Gynecology Hospital Affiliated to Fudan University | 1. https://mp.weixin.qq.com/s/gZy0IAByJ6dj1meRjxHGpQ |
| Digital Intelligent Medical Research Platform at Peking University First Hospital | 1. https://www.pkufh.com/Html/News/Articles/60014.html |
| “CHANGE” at Children's Hospital Zhejiang University School of Medicine | 1. https://mp.weixin.qq.com/s/INMK15P3FxM3YtrSbAiouA  2. https://mp.weixin.qq.com/s/sDsKtc9V8-cRFPtS91tsQQ |
| DeepSeek at The First Affiliated Hospital of Anhui Medical University | 1. https://www.ayfy.com/info/1203/26261.htm  2. https://www.ayfy.com/info/1178/27010.htm |
| “DeepSeek × Wudarenyi” at Renmin Hospital of Wuhan University | 1. https://mp.weixin.qq.com/s/bySwDKyz-zTmtlseIGUmKA  2. https://mp.weixin.qq.com/s/ldxXvFfm4N4nKwtWDQgkHw |

| **Hospital deployed models** | **Information sources** |
| --- | --- |
| DeepSeek at Jiangsu Provincial People's Hospital | 1. https://www.jsph.org.cn/info/1671/149921.htm |
| DeepSeek at Qilu Hospital of Shandong University | 1. https://www.qiluhospital.com/show-25-39572-1.html  2. https://www.qiluhospital.com/show-25-39752-1.html |
| DeepSeek at The Affiliated Hospital of Qingdao University | 1. https://mp.weixin.qq.com/s/KBABFggPvh-bc3MNasdi2A  2. https://www.hit180.com/71828.html |
| DeepSeek at The First Hospital of Jilin University | 1. https://www.jdyy.cn/index.php?m=home&c=View&a=index&aid=77922  2. https://www.jdyy.cn/index.php?m=home&c=View&a=index&aid=79352  3. https://jdyy.cn/index.php?m=home&c=View&a=index&aid=77930  4. https://jdyy.cn/index.php?m=home&c=View&a=index&aid=79140 |
| Innovative Drug Clinical Application Intelligent Decision System (IDICDS) at Peking University First Hospital | 1. https://www.pkufh.com/Html/News/Articles/60038.html |
| DeepSeek at Sichuan Provincial People's Hospital | 1. https://www.samsph.cn/news_major/2025/7axwJJey.html  2. https://article.xuexi.cn/articles/index.html?art_id=87940193581932920  51&cdn=https%3A%2F%2Fregion-sichuan-resource&item_id=8794019358193292051  3. https://kscgc.scgchc.com/sctv/h5/v7/newsShare.html?id=18975455076  73980930&appUserId=486762  4. https://www.samsph.cn/news_hos/2025/Xe0PKKex.html |
| DeepSeek at Zhongnan Hospital of Wuhan University | 1. https://www.znhospital.cn/detail/14666.html  2. https://m.hbtv.com.cn/cmdetail/527393 |
| DeepSeek at Tianjin Medical University General Hospital | 1. https://mp.weixin.qq.com/s/h7w_7A0pSSCLqhCwvncBlQ  2. https://mp.weixin.qq.com/s/yetyIQ8MgVAzTNXwO4EyXg  3. http://tj.people.com.cn/n2/2025/0226/c408648-41147296.html |
| “Zheng Xiao Yi” at The First Affiliated Hospital of Zhengzhou University | 1. https://www.zdyfy.com/yydt/zhxw/content_32220  2. https://mp.weixin.qq.com/s?__biz=MzU5MTU2NTgyMQ==&mid=22474  84277&idx=1&sn=904762321b171674f634f7036c7444fe&chksm=fe2c45e7c95bccf1faebcfd1e96a4c18df0d5f31ab50888518bc4cf4e91605adc62defac7ded&scene=27 |
| **Hospital deployed models** | **Information sources** |
| Pancreatitis DeepSeek Large Language Model Integrated Traditional Chinese and Western Medicine at West China Hospital of Sichuan University | 1. https://www.wchscu.cn/public/affairs/rewards3/84703.html |
| "AI-assisted Rare Disease Precision Diagnosis Platform" at Sichuan Provincial People's Hospital | 1. https://www.samsph.cn/news_major/2025/7axwJJey.html  2. https://mp.weixin.qq.com/s/huw3wDk6lFF_oOVwU-wIig  3. https://mp.weixin.qq.com/s/S-F5VpbB2N07EBSbhOaVRA |
| DeepSeek at The Second Affiliated Hospital of Army Medical University | 1. https://mp.weixin.qq.com/s/mJrhydyDLUFIH_lwIKn1KQ |
| Multi-scene Medical Intelligent Agent at Drum Tower Hospital of Medical School of Nanjing University | 1. https://www.njglyy.com/search/detail.aspx?Id=12055&mtt=1  2. https://www.njdaily.cn/news/2025/0313/7437769070497915229.html |
| DeepSeek at Zhongda Hospital of Southeast University | 1. https://www.njzdyy.com/info/1011/9666.htm |
| DeepSeek at Union Hospital of Tongji Medical College, Huazhong University of Science and Technology | 1. https://www.tjh.com.cn/TjhNews/20250228_54182.html  2. https://news.hubeidaily.net/hbrbsharenew/news_detail/5/3687583/33  37309/0?w=1740649495531&uik=7Hy68ffD&share_plat=wechat&sec=c1ae39fa&contentType=5&historyback=1 |
| DeepSeek at Shengjing Hospital of China Medical University | 1. https://m.baidu.com/bh/m/detail/ar_9496334916645083167  2. https://mp.weixin.qq.com/s?__biz=MzA4NTkyODA1Ng==&mid=265057  2038&idx=7&sn=3b0db3bac15288bff07846eb654b19ca&chksm=862a2ca96824a4239dca338ed11409da192424439ed70639e88b9e13d2ff3d9c9a125ced4fea&scene=27  3. https://mp.weixin.qq.com/s/fqJFlD5XfehQjZ7q9qYuqw |
| DeepSeek at Peking University Third Hospital | 1. https://www.puh3.net.cn/info/2001/148601.htm  2. https://www.puh3.net.cn/info/1971/147991.htm |
| Tongji Hospital Large Language Model at Tongji Hospital of Tongji Medical College, Huazhong University of Science and Technology | 1. https://www.whuh.com/info/1055/58805.htm  2. https://mp.weixin.qq.com/s/W6WhdMv58ZtEftsAkuYr_A |
| DeepSeek at Shandong Provincial Hospital of Shandong First Medical University | 1. https://www.sph.com.cn/Html/News/Articles/33798.html?WebVisitSh  ield=S5zyEBWSrBbqaAylSufz  2. https://www.sph.com.cn/Html/News/Articles/33910.html?WebVisitShi  eld=G7dv265A2T05CaxMk7Vp |

| **Hospital deployed models** | **Information sources** |
| --- | --- |
| DeepSeek at Guangzhou Women and Children's Medical Center | 1. https://www.gzfezx.com/news/show/10063.html  2. https://baijiahao.baidu.com/s?id=1825744812942506403&wfr=spider&f  or=pc |
| DeepSeek at The First Affiliated Hospital of Harbin Medical University | 1. https://www.54dr.org.cn/news/944.html  2. https://www.54dr.org.cn/news/946.html |
| DeepSeek at Beijing Shijitan Hospital of Capital Medical University | 1. https://www.bjsjth.cn/Html/News/Articles/15073295.html  2. https://www.hit180.com/73389.html |
| “Xiehe Zhishu” at Peking Union Medical College Hospital | 1. https://www.pumch.cn/detail/40419.html |
| DeepSeek at Shenzhen People's Hospital | 1. https://www.szhospital.com/?fid=search&part=localfrm&type_code=&p  k=18755&qstr=DeepSeek |
| “Xiao Ya Xi" at Xiangya Hospital of Central South University | 1. https://mp.weixin.qq.com/s/Nk-sAnlU7GO1Q2fGy1-9Gw |
| Intelligent Tumor Diagnosis and Treatment at Shanghai Cancer Center of Fudan University | 1. https://www.shca.org.cn/Home/news/content/id/307/pid/26485  2. https://mp.weixin.qq.com/s/SYh8YmgGZCdKZgeJ6a0DWg |
| DeepSeek at The First Affiliated Hospital of University of Science and Technology of China | 1. https://www.ahslyy.com.cn/cn/News/info_94_itemid_100643.html  2. https://wjw.ah.gov.cn/xwzx/mtjj/57706621.html |
| “DS-Dr. Xiaobu” at Children's Hospital of Fudan University | 1. https://ch.shmu.edu.cn/main/news/content/id/492/pid/22955.html |
| DeepSeek at Beijing Anzhen Hospital of Capital Medical University | 1. https://baijiahao.baidu.com/s?id=1826293240642235811&wfr=spider&f  or=pc  2. https://mp.weixin.qq.com/s/ymNFLO5pcO9U-WWzsMH0wA |
| DeepSeek at Beijing Jishuitan Hospital of Capital Medical University | 1. https://www.jst-hosp.com.cn/Html/News/Articles/43890.html |
| DeepSeek at Henan Provincial People's Hospital | 1. https://mp.weixin.qq.com/s/ZBvpOR58B0iWD3pJuKE38A  2. https://wap.zhengguannews.cn/html/zgh/319620.html |
| “Xiaoganren” at Zhongda Hospital of Southeast University | 1. https://www.njzdyy.com/info/1011/9695.htm |
| Surgical Risk AI Large Language Model at Shandong Provincial Hospital Affiliated to Shandong First Medical University | 1.https://www.sph.com.cn/Html/News/Articles/33910.html?WebVisitShie  ld=G7dv265A2T05CaxMk7Vp |

| **Hospital deployed models** | **Information sources** |
| --- | --- |
| DeepSeek at The First Affiliated Hospital of Nanchang University | 1. https://mp.weixin.qq.com/s/zSpg8XD_4GNW4G3lroQAEw  2. https://mp.weixin.qq.com/s/aVfw3RYEsjQ--T0LhJQbAA  3. https://mp.weixin.qq.com/s/aVfw3RYEsjQ--T0LhJQbAA  4. https://mp.weixin.qq.com/s/5PeR2HhsLLu63_IXtEN8Gw  5. https://mp.weixin.qq.com/s/sVKAepC11WTYmywJaTv-6g |
| Peritoneal Dialysis Treatment Large Language Model at The First Affiliated Hospital of Sun Yat-sen University | 1. https://mp.weixin.qq.com/s/BoeiywubAtrHSfzX_r-uYw |
| West China Digital Medicine Large Language Model at West China Second University Hospital of Sichuan University | 1. https://mp.weixin.qq.com/s/6Y142nQTEQkMc8JelQc-Iw  2. https://mp.weixin.qq.com/s/8heaTI4cNXdr0UB6vBAGZg |
| DeepSeek at Women's Hospital of School of Medicine, Zhejiang University | 1. https://mp.weixin.qq.com/s/0-Y0UL_RVmYbbWDRyYVfXg |
| DeepSeek at Union Hospital of Fujian Medical University | 1. http://www.fjxiehe.com/xwzx/zhxw/202503/t20250327_16463.htm |
| DeepSeek at The Second Affiliated Hospital of Harbin Medical University | 1. https://www.hrbmush.edu.cn/info/1042/8626.htm  2. https://mp.weixin.qq.com/s/dR9wOXW2srlXvmM6o0C0IQ |
| DeepSeek at Zhujiang Hospital of Southern Medical University | 1. https://www.zjyy.com.cn/template/hostpital/news_details.html?newsI  d=12561  2. https://www.zjyy.com.cn/template/hostpital/news_details.html?newsI  d=12709 |
| DeepSeek at Beijing Friendship Hospital of Capital Medical University | 1. https://mp.weixin.qq.com/s?__biz=MzA5Mjg2Mjg3NA==&mid=2703157  597&idx=2&sn=a7eef51c6278826573deafdf99ba1d39&chksm=b5cd57596d62da5c1ba33c6e3a86648d476eec316bccc9c75b963eef3c4ad01ff47e8e7d16d5&scene=27 |
| “Shenong-Shuwen” at Tongji Hospital of Tongji Medical College, Huazhong University of Science and Technology | 1. https://jms.ctdsb.net/jmythshare/#/news_detail?contentType=5&conte  ntId=2433829&cId=0&uik=CMlnURnm&share_plat=wechat&sec=bada7a4e  2. https://www.tjh.com.cn/TjhNews/20250417_54580.html |
| DeepSeek at The First Affiliated Hospital of Fujian Medical University | 1. https://mp.weixin.qq.com/s/g5LfbC_vH1sYNz20UXS2vA  2. https://mp.weixin.qq.com/s/fCE_d4qdoABXchBNMtxs_A  3. https://mp.weixin.qq.com/s/237K08SqcMCnZZgBRobhAg |

| **Hospital deployed models** | **Information sources** |
| --- | --- |
| “Yaoxiaosu” at The First Affiliated Hospital of Soochow University | 1. https://app.suzhou-news.cn/news/300718538  2. https://mp.weixin.qq.com/s?__biz=MzA4NzUzNzkzMQ==&mid=2650464  351&idx=2&sn=7cd4e99faa3c8c39335db35bfadea62e&chksm=89cde8993036920d7447896a0d3aeb03a209623f8ea1dd6676d3c4bdbf4ca45efd8417e26085&scene=27 |
| “Tongji Mulan” at Tongji Hospital of Tongji Medical College, Huazhong University of Science and Technology | 1. https://mp.weixin.qq.com/s/FgKpIouPzcHFlINOQRyXGg |
| Chronic Kidney Disease Comprehensive Management Large Language Model at Nanfang Hospital of Southern Medical University | 1. https://news.smu.edu.cn/info/1016/115726.htm  2. https://content-static.cctvnews.cctv.com/snow-book/index.html?item_i  d=2713572931152225963&track_id=B51ACECE-DF16-4C2F-80B9-BD86D6B63D2F_769574989121 |
| “Aixin Zhihu” at Guangzhou Women and Children's Medical Center | 1. https://mp.weixin.qq.com/s/U2tzI5O_uwtHStcoujDLVQ  2. https://mp.weixin.qq.com/s/pXe6_Hpil9Whn69EHoGJZw |
| “Lung Smart” at Shanghai Pulmonary Hospital | 1. https://baijiahao.baidu.com/s?id=1833365087156687203&wfr=spider&f  or=pc  2. https://mp.weixin.qq.com/s/-T-OD9ZEmiRVlGV-chW_3Q  3. https://mp.weixin.qq.com/s/xuf4kMwQSLS6uLjQ5yvZ_w |

**Table S3. Characteristics of hospital deployed DeepSeek (n=58, chronological order)**

| **Hospital deployed DeepSeek** | **Province** | **Version** | **Deployment Date** | **Deployment type** | **Deployment specialty** | **Application scenarios** |
| --- | --- | --- | --- | --- | --- | --- |
| DeepSeek at The Third Affiliated Hospital of Sun Yat-sen University | Guangdong | R1 | 10-Feb-25 | Private deployment | Not specify | Diagnosis formulation;  Clinical documentation;  Literature synthesis |
| DeepSeek at Shanghai Sixth People's Hospital | Shanghai | Not specify | 13-Feb-25 | Private deployment | Not specify | Clinical Documentation;  Hospital administration support;  Appointment coordination |
| Qilu Clinical Skills Large Model at Qilu Hospital of Shandong University | Shandong | R1 | 14-Feb-25 | Not specify | Not specify | Training medical trainee |
| DeepSeek at Nanfang Hospital of Southern Medical University | Guangdong | R1 | 17-Feb-25 | Private deployment | Not specify | Diagnosis formulation;  Clinical documentation |
| “Xiehe Taichu” at Peking Union Medical College Hospital | Beijing | R1 | 19-Feb-25 | Not specify | Rare diseases | Diagnosis formulation |
| “Xiaohong AI” Patient Assistant at Obstetrics and Gynecology Hospital Affiliated to Fudan University | Shanghai | R1 | 19-Feb-25 | Not specify | Not specify | Appointment coordination |
| Digital Intelligent Medical Research Platform at Peking University First Hospital | Beijing | R1 | 21-Feb-25 | Private deployment | Not specify | Literature synthesis;  Training medical trainee |
| “CHANGE” at Children's Hospital Zhejiang University School of Medicine | Zhejiang | Not specify | 21-Feb-25 | Not specify | Pediatrics | Diagnosis formulation;  Treatment and medication recommendation |
| DeepSeek at The First Affiliated Hospital of Anhui Medical University | Anhui | R1 | 21-Feb-25 | Private deployment | Not specify | Diagnosis formulation |
| “DeepSeek × Wudarenyi” at Renmin Hospital of Wuhan University | Hubei | R1 | 22-Feb-25 | Private deployment | Not specify | Clinical Documentation;  Appointment coordination;  Literature synthesis;  Research protocol support |
| DeepSeek at Jiangsu Provincial People's Hospital | Jiangsu | Not specify | 23-Feb-25 | Not specify | Not specify | Diagnosis formulation;  Treatment and medication recommendation;  Hospital administration support;  Appointment coordination |
| DeepSeek at Qilu Hospital of Shandong University | Shandong | R1 | 23-Feb-25 | Private & cloud-based deployment | Not specify | Clinical Documentation;  Hospital administration support;  Appointment coordination |
| DeepSeek at The Affiliated Hospital of Qingdao University | Shandong | R1 | 23-Feb-25 | Private deployment | Not specify | Clinical Documentation;  Hospital administration support;  Appointment coordination |
| DeepSeek at The First Hospital of Jilin University | Jilin | R1 | 23-Feb-25 | Private deployment | Not specify | Diagnosis formulation;  Clinical documentation;  Hospital administration support |
| Innovative Drug Clinical Application Intelligent Decision System (IDICDS) at Peking University First Hospital | Beijing | R1 | 24-Feb-25 | Private deployment | Oncology | Diagnosis formulation;  Treatment and medication recommendation;  Clinical documentation;  Research protocol support |
| DeepSeek at Sichuan Provincial People's Hospital | Sichuan | Not specify | 24-Feb-25 | Private deployment | Not specify | Diagnosis formulation;  Treatment and medication recommendation;  Clinical documentation;  Hospital administration support;  Appointment coordination |
| DeepSeek at Zhongnan Hospital of Wuhan University | Hubei | Not specify | 24-Feb-25 | Private deployment | Not specify | Treatment and medication recommendation;  Clinical Documentation;  Hospital administration support |
| DeepSeek at Tianjin Medical University General Hospital | Tianjin | Not specify | 24-Feb-25 | Not specify | Not specify | Appointment coordination |
| “Zheng Xiao Yi” at The First Affiliated Hospital of Zhengzhou University | Henan | Not specify | 25-Feb-25 | Not specify | Not specify | Appointment coordination |
| Pancreatitis DeepSeek Large Language Model Integrated Traditional Chinese and Western Medicine at West China Hospital of Sichuan University | Sichuan | Not specify | 25-Feb-25 | Private deployment | Gastroenterology | Diagnosis formulation;  Treatment and medication recommendation |
| "AI-assisted Rare Disease Precision Diagnosis Platform" at Sichuan Provincial People's Hospital | Sichuan | Not specify | 25-Feb-25 | Not specific | Rare diseases | Diagnosis formulation;  Treatment and medication recommendation |
| DeepSeek at The Second Affiliated Hospital of Army Medical University | Chongqing | R1 | 25-Feb-25 | Private deployment | Not specify | Diagnosis formulation;  Treatment and medication recommendation;  Clinical documentation;  Research protocol support |
| Multi-scene Medical Intelligent Agent at Drum Tower Hospital of Medical School of Nanjing University | Jiangsu | Not specify | 26-Feb-25 | Private deployment | Not specify | Diagnosis formulation;  Treatment and medication recommendation;  Clinical documentation;  Appointment coordination;  Training medical trainee |
| DeepSeek at Zhongda Hospital of Southeast University | Jiangsu | R1 | 26-Feb-25 | Private deployment | Not specify | Clinical Documentation;  Appointment coordination |
| DeepSeek at Union Hospital of Tongji Medical College, Huazhong University of Science and Technology | Hubei | R1 | 27-Feb-25 | Private deployment | Not specify | Diagnosis formulation;  Treatment and medication recommendation;  Clinical documentation |
| DeepSeek at Shengjing Hospital of China Medical University | Liaoning | Not specify | 27-Feb-25 | Private deployment | Not specify | Clinical Documentation;  Hospital administration support;  Appointment coordination |
| DeepSeek at Peking University Third Hospital | Beijing | R1 | 28-Feb-25 | Private deployment | Not specify | diagnosis formulation;  Treatment and medication recommendation;  Clinical documentation |
| Tongji Hospital Large Language Model at Tongji Hospital of Tongji Medical College, Huazhong University of Science and Technology | Hubei | Not specify | 28-Feb-25 | Private deployment | Not specify | Clinical Documentation;  Appointment coordination |
| DeepSeek at Shandong Provincial Hospital of Shandong First Medical University | Shandong | R1 | 3-Mar-25 | Not specify | Not specify | Diagnosis formulation;  Patient education;  Treatment adherence support;  Appointment coordination;  Literature synthesis;  Research protocol support |
| DeepSeek at Guangzhou Women and Children's Medical Center | Guangdong | R1 | 3-Mar-25 | Private deployment | Not specify | Clinical Documentation;  Appointment coordination |
| DeepSeek at The First Affiliated Hospital of Harbin Medical University | Heilongjiang | Not specify | 3-Mar-25 | Not specify | Not specify | Clinical Documentation;  Appointment coordination |
| DeepSeek at Beijing Shijitan Hospital of Capital Medical University | Beijing | Not specify | 5-Mar-25 | Private deployment | Not specify | Diagnosis formulation;  Clinical documentation;  Hospital administration support |
| “Xiehe Zhishu” at Peking Union Medical College Hospital | Beijing | R1 | 6-Mar-25 | Not specify | Not specify | Diagnosis formulation;  Clinical documentation;  Hospital administration support;  Treatment adherence support;  Appointment coordination |
| DeepSeek at Shenzhen People's Hospital | Guangdong | R1 | 6-Mar-25 | Private deployment | Not specify | Diagnosis formulation;  Appointment coordination |
| “Xiao Ya Xi" at Xiangya Hospital of Central South University | Hunan | Not specify | 7-Mar-25 | Private deployment | Not specify | Appointment coordination |
| Intelligent Tumor Diagnosis and Treatment at Shanghai Cancer Center of Fudan University | Shanghai | R1 | 8-Mar-25 | Private deployment | Oncology | Diagnosis formulation;  Treatment and medication recommendation;  Clinical documentation;  Treatment adherence support;  Appointment coordination |
| DeepSeek at The First Affiliated Hospital of University of Science and Technology of China | Anhui | R1 | 9-Mar-25 | Private deployment | Not specify | Diagnosis formulation |
| “DS-Dr. Xiaobu” at Children's Hospital of Fudan University | Shanghai | Not specify | 10-Mar-25 | Private deployment | Pediatrics | Diagnosis formulation;  Treatment and medication recommendation;  Hospital administration support;  Appointment coordination |
| DeepSeek at Beijing Anzhen Hospital of Capital Medical University | Beijing | R1 | 11-Mar-25 | Private deployment | Not specify | Clinical Documentation |
| DeepSeek at Beijing Jishuitan Hospital of Capital Medical University | Beijing | Not specify | 17-Mar-25 | Private deployment | Cardiology | Diagnosis formulation;  Treatment and medication recommendation;  Clinical documentation |
| DeepSeek at Henan Provincial People's Hospital | Henan | R1 | 18-Mar-25 | Private deployment | Respiratory and critical care medicine | Diagnosis formulation |
| “Xiaoganren” at Zhongda Hospital of Southeast University | Jiangsu | Not specify | 19-Mar-25 | Not specify | Oncology | Diagnosis formulation;  Treatment and medication recommendation |
| Surgical Risk AI Large Language Model at Shandong Provincial Hospital Affiliated to Shandong First Medical University | Shandong | Not specify | 20-Mar-25 | Not specify | Surgery | Diagnosis formulation;  Treatment and medication recommendation |
| DeepSeek at The First Affiliated Hospital of Nanchang University | Jiangxi | Not specify | 21-Mar-25 | Private deployment | Not specify | Diagnosis formulation;  Clinical documentation;  Hospital administration support;  Appointment coordination |
| Peritoneal Dialysis Treatment Large Language Model at The First Affiliated Hospital of Sun Yat-sen University | Guangdong | Not specify | 23-Mar-25 | Private deployment | Urology | Diagnosis formulation;  Treatment and medication recommendation;  Patient education;  Treatment adherence support |
| West China Digital Medicine Large Language Model at West China Second University Hospital of Sichuan University | Sichuan | R1 | 26-Mar-25 | Private deployment | Not specify | Diagnosis formulation;  Treatment and medication recommendation;  Appointment coordination |
| DeepSeek at Women's Hospital of School of Medicine, Zhejiang University | Zhejiang | Not specify | 27-Mar-25 | Not specify | Not specify | Patient education;  Treatment adherence support;  Appointment coordination |
| DeepSeek at Union Hospital of Fujian Medical University | Fujian | R1 | 27-Mar-25 | Private deployment | Not specify | Diagnosis formulation;  Treatment and medication recommendation;  Clinical documentation;  Hospital administration support;  Appointment coordination |
| DeepSeek at The Second Affiliated Hospital of Harbin Medical University | Heilongjiang | Not specify | 3-Apr-25 | Not specify | Not specify | Appointment coordination;  Literature synthesis |
| DeepSeek at Zhujiang Hospital of Southern Medical University | Guangdong | Not specify | 17-Apr-25 | Not specify | Not specify | Clinical Documentation;  Appointment coordination |
| DeepSeek at Beijing Friendship Hospital of Capital Medical University | Beijing | Not specify | 27-Apr-25 | Not specify | Chinese traditional medicine | Not specified |
| “Shenong-Shuwen” at Tongji Hospital of Tongji Medical College, Huazhong University of Science and Technology | Hubei | Not specify | 30-Apr-25 | Private deployment | Anesthesiology | Diagnosis formulation;  Treatment and medication recommendation |
| DeepSeek at The First Affiliated Hospital of Fujian Medical University | Fujian | Not specify | 8-May-25 | Private deployment | Not specify | Clinical Documentation;  Appointment coordination |
| “Yaoxiaosu” at The First Affiliated Hospital of Soochow University | Jiangsu | Not specify | 14-May-25 | Not specify | Not specify | Patient education;  Treatment adherence support |
| “Tongji Mulan” at Tongji Hospital of Tongji Medical College, Huazhong University of Science and Technology | Hubei | Not specify | 16-May-25 | Private deployment | Oncology | Diagnosis formulation;  Patient education;  Treatment adherence support;  Health monitoring |
| Chronic Kidney Disease Comprehensive Management Large Language Model at Nanfang Hospital of Southern Medical University | Guangdong | Not specify | 19-May-25 | Not specify | Urology | Diagnosis formulation;  Treatment and medication recommendation;  Patient education;  Treatment adherence support |
| “Aixin Zhihu” at Guangzhou Women and Children's Medical Center | Guangdong | Not specify | 22-May-25 | Not specify | Not specify | Diagnosis formulation;  Treatment and medication recommendation |
| “Lung Smart” at Shanghai Pulmonary Hospital | Shanghai | Not specify | 28-May-25 | Not specify | Respiratory and critical care medicine | Diagnosis formulation;  Treatment and medication recommendation;  Clinical documentation |

R1 = DeepSeek-reasoner.

The table presents characteristics of deployed DeepSeek of the top 100 hospitals in China in chronological order based on deployment date. The 100 hospitals were selected based on China’s hospital rankings (2023). We retrieved information related to DeepSeek deployment from the top 100 Chinese hospitals’ official websites and WeChat accounts. Deployment date was determined by the date of hospital official disclosure of DeepSeek deployment. The data was updated to 28 June, 2025 (date of last search).

**Table S4. Characteristics of studies assessing DeepSeek use in health care (n=27, chronological order)**

| **Author** | **Date** | **DeepSeek Version** | **Intended use specialty** | **Specific tasks** | **Application scenarios** |
| --- | --- | --- | --- | --- | --- |
| Ishith Seth | Feb-25 | V3 | Surgery | To provide management strategies for Dupuytren’s disease | Treatment and medication recommendation |
| Gianluca Marcaccini | Mar-25 | V3 | Surgery | To provide management strategies for hand fractures | Treatment and medication recommendation |
| Gianluca Marcaccini | Apr-25 | V3 | Surgery | To provide rehabilitation programs for head and neck surgery | Treatment and medication recommendation |
| Ömer Faruk Kaygisiz | Apr-25 | V3 | Dentistry | To diagnose oral lesions | Diagnosis formulation |
| Xiangming Cai | Apr-25 | R1 | General | To screen literature for meta-analyses | Literature synthesis |
| Apostolos Mavridis | Apr-25 | R1 | General | To map medical ontology for knowledge graphs | Clinical knowledge assessment |
| Sarah Sandmann | Apr-25 | V3; R1 | General | To provide diagnosis and treatment recommendation | Diagnosis formulation;  Treatment and medication recommendation |
| Mohammad Javed Ali | May-25 | Not specify | Ophthalmology | To generate education resources for patients and physicians for lacrimal drainage disorders | Patient education;  Medical education |
| Peng-Wei Luo | May-25 | R1 | Oncology | To answer questions related to prostate cancer radiotherapy | Patient education;  Medical education |
| Soumil Prasad | May-25 | R1 | Otolaryngology | To answer questions related to otolaryngology procedures | Patient education |
| Ren-Chun Du | May-25 | V3; R1 | Gastroenterology | To answer questions related to Helicobacter pylori | Patient education;  Medical education |
| Cheng Jiao | May-25 | R1 | Ophthalmology | To diagnosis for corneal disease | Diagnosis formulation |
| Hasaam Uldin | May-25 | R1 | Radiology | To answer questions related to musculoskeletal radiology | Patient education;  Medical education |
| Luxiang Shang | May-25 | R1 | Multidisciplinary Clinic | To answer questions related to cardiovascular-kidney-metabolic syndrome | Patient education;  Medical education |
| Rong Chen | May-25 | Not specify | Rehabilitation | To answer questions related to low back pain | Patient education;  Medical education |
| Jiayi Dai | May-25 | R1 | Gastroenterology | To classify Crohn’s disease based on computed tomography enterography reports | Diagnosis formulation |
| Daniel Spitzl | May-25 | V3 | Hepatology | To classify liver lesions from magnetic resonance imaging reports | Diagnosis formulation |
| Kai Wang | May-25 | R1 | Surgery | To predict cardiopulmonary bypass-associated acute kidney injury | Health monitoring |
| Tuğgen Özcivelek | May-25 | R1 | Surgery | To answer questions related to dental and maxillofacial prostheses | Patient education |
| Mi Zhou | Jun-25 | V3; R1 | Surgery | To generate patient education materials for spinal surgeries | Patient education |
| Junyang Ma | Jun-25 | R1 | Anesthesiology | To assist physician decision-making for progressive anesthesia crisis | Treatment and medication recommendation |
| Olivier Niel | Jun-25 | V3 | Pediatric | To assist diagnostic formulation, treatment modification, and prognostic assessment in pediatric nephrology | Diagnosis formulation;  Treatment and medication recommendation |
| Hilmi Anil Dincer | Jun-25 | V3 | Surgery | To answer questions related to laparoscopic cholecystectomy | Patient education |
| Xintong Wu | Jun-25 | R1 | General | To diagnose complex cases | Diagnosis formulation |
| Serhat Gurbuz | Jun-25 | Not specify | Surgery | To answer questions related to total knee arthroplasty of gonarthrosis patients | Patient education |
| Onur Gültekin | Jun-25 | R1 | Surgery | To support evidence-based patient education for anterior cruciate ligament surgery | Patient education |
| Burcu Vural Camalan | Jun-25 | V3 | Oncology | To classify stages of head and neck cancers and give treatment recommendation | Diagnosis formulation;  Treatment and medication recommendation |

V3 = DeepSeek-Chat; R1 = DeepSeek-reasoner.

The table presents characteristics of studies evaluating DeepSeek use in health care. Studes were ordered in chronological order based on their publication month. Studies were retrieved from PubMed and Web of Science which were published between 15 Jan, 2025 (release date of DeepSeek) and 28 June, 2025 (date of last search).

**Table S5.** **Results of hospital disclosure of deployed DeepSeek performance (n = 13)**

| **Hospital deployed DeepSeek** | **Evaluation dimension** | **Evaluation indicators** | **Performance** |
| --- | --- | --- | --- |
| DeepSeek at Nanfang Hospital of Southern Medical University | Accuracy | Abnormal patient test results interpretation | 98% |
| “CHANGE” at Children's Hospital Zhejiang University School of Medicine | Accuracy | Congenital heart disease diagnosis | 95% |
| DeepSeek at The First Hospital of Jilin University | Deployment metrics | Response times | <3 seconds |
| Pancreatitis DeepSeek Large Language Model Integrated Traditional Chinese and Western Medicine at West China Hospital of Sichuan University | Accuracy | Pancreatitis treatment recommendation | >90% |
| "AI-assisted Rare Disease Precision Diagnosis Platform" at Sichuan Provincial People's Hospital | Accuracy | Myasthenia gravis and cardiac amyloidosis prediction | >90% |
| Multi-scene Medical Intelligent Agent at Drum Tower Hospital of Medical School of Nanjing University | Accuracy | Patient triage | 86% |
| DeepSeek at Union Hospital of Tongji Medical College, Huazhong University of Science and Technology | Accuracy | Rare diseases diagnosis | 91.20% |
| DeepSeek at Guangzhou Women and Children's Medical Center | Accuracy | Patient information classification | >90% |
| DeepSeek at Shenzhen People's Hospital | Accuracy | Negative pathological section | 100% |
| “DS-Dr. Xiaobu” at Children's Hospital of Fudan University | Accuracy | Complex cases diagnosis | >95% |
| West China Digital Medicine Large Language Model at West China Second University Hospital of Sichuan University | Accuracy | Diagnosis and treatment recommendation | >85% |
| “Shenong-Shuwen” at Tongji Hospital of Tongji Medical College, Huazhong University of Science and Technology | Accuracy | Perioperative patient follow-up summary | 97.50% |
| “Lung Smart” at Shanghai Pulmonary Hospital | Accuracy | Treatment recommendation for lung diseases | >95% |

NI = No information.

The table presents results of hospital disclosure of DeepSeek’s performance (n=13). For the rest, 8 of them indicate a pre-deployment evaluation but do not present their results and others do not report relevant information. DeepSeek models were presented in chronological order based on deployment date to be consistent with table 1.

**Table S6. Performance and risks of DeepSeek in health care based on studies (n=27, chronological order)**

| **Author** | **Date** | **Evaluation cases** | **Case number** | **Evaluation dimension** | **Evaluation approach** | **Evaluation indicators** | **Performance** | **Risks** |
| --- | --- | --- | --- | --- | --- | --- | --- | --- |
| Ishith Seth | Feb-25 | Simulated cases | 21 | Accuracy | Expert rating | Accuracy;  Precision;  Recall;  F1-score | Accuracy: 63.6%;  Precision: 55.0%;  Recall: 52.0%;  F1-score: 53.5% | Inappropriate recommendation |
| Gianluca Marcaccini | Mar-25 | Real-world cases | 58 | Accuracy | Expert rating | Accuracy;  Precision;  Recall;  F1-score | Accuracy: 63.79%;  Precision: 61.17%;  Recall: 57.89%;  F1-score: 59.52% | NI |
| Gianluca Marcaccini | Apr-25 | Simulated cases | 10 | Accuracy;  Comprehensiveness;  Fairness | Expert rating;  Text assessment | Overall quality;  DISCERN;  Readability | Overall quality: 4.00±0.82 (range: 1-5);  DISCERN: 46.90±2.60 (range: 0-100);  FRES: 3.05±4.78 (range: 0-100);  FKGL: 20.15 ± 1.53 (educational grade);  CLI: 19.29±1.39 (educational grade) | Inappropriate recommendation; misaligned content to patient needs and conditions |
| Ömer Faruk Kaygisiz | Apr-25 | Simulated cases | 16 | Accuracy;  Factuality | Expert rating | Overall quality;  Fake citation | Overall quality: 4.02±0.36 (range: 1-5);  Fake citations: 16.7% (8/48) | Fake citation |
| Xiangming Cai | Apr-25 | Real-world cases | 4 | Accuracy | Ground truth comparison | Precision;  Recall;  F1-score | Precision: 72.1%-100%;  Recall: 58.4%-94.6%;  F1-score: 58.4%-87.6% | NI |
| Apostolos Mavridis | Apr-25 | Simulated cases | 108 | Accuracy | Ground truth comparison | Precision;  Recall;  F1-score | Precision: 25.76%;  Recall: 32.69%;  F1-score: 28.81% | NI |
| Sarah Sandmann | Apr-25 | Simulated cases | 125 | Accuracy;  Comprehensiveness | Expert rating | Overall quality | V3: diagnosis: 4.69 (range: 1-5);  R1: diagnosis: 4.70 (range: 1-5); treatment: 4.48 (range: 1-5) | Inappropriate recommendation |
| Mohammad Javed Ali | May-25 | Simulated questions | 25 | Accuracy | Expert rating | Overall quality | Correct: 60%;  Partially correct: 36%;  Factually incorrect: 4% | Inappropriate recommendation |
| **Author** | **Date** | **Evaluation cases** | **Case number** | **Evaluation dimension** | **Evaluation approach** | **Evaluation indicators** | **Performance** | **Risks** |
| Peng-Wei Luo | May-25 | Simulated questions | 44 | Accuracy;  Comprehensiveness | Expert rating | Overall quality | Chinese scenario (range 1-5): 75.76% (scored 5); 12.12% (scored 4); 12.12% (scored 3);  English scenario (range 1-5): 54.55% (scored 5); 27.27% (scored 4); 15.15% (scored 3); 3.03% (scored 2) | NI |
| Soumil Prasad | May-25 | Simulated questions | 5 | Accuracy;  Comprehensiveness | Expert rating | Qualitative summary of overall quality | Concise responses that were clinically relevant and clear | Inappropriate recommendation |
| Ren-Chun Du | May-25 | Simulated questions | 21 | Accuracy | Expert rating | Accuracy | V3: 87.0% - 96.2%;  R1: 94.0% - 96.2% | NI |
| Cheng Jiao | May-25 | Simulated cases | 20 | Accuracy | Ground truth comparison | Overall accuracy | Overall: 65%; | NI |
| Hasaam Uldin | May-25 | Simulated questions | 10 | Accuracy | Expert rating | Overall quality | Overall quality: 2.9 (range: 1-5) | hallucination; fake citation |
| Luxiang Shang | May-25 | Simulated questions | 10 | Accuracy;  Comprehensiveness | Expert rating | Overall quality | Overall quality: 86.00±2.83 (range: 20-100) | inappropriate recommendation; outdated knowledge |
| Rong Chen | May-25 | Simulated questions | 80 | Accuracy;  Comprehensiveness | Expert rating | Overall quality;  Accuracy;  Relevance;  Clarity;  Benefit;  Completeness | Overall quality: 3.81 (range: 1-5);  Accuracy: 3.83 (range: 1-5);  Relevance: 3.74 (range: 1-5);  Clarity: 3.85 (range: 1-5);  Benefit: 3.86 (range: 1-5);  Completeness: 3.77 (range: 1-5) | NI |
| Jiayi Dai | May-25 | Real-world cases | 198 | Accuracy | Ground truth comparison | Accuracy;  Precision;  Recall;  F1-score | Accuracy: 88.9%;  Precision: 96.8%;  Recall: 79.7%;  F1-score: 87.4% | Inappropriate recommendation |

| **Author** | **Date** | **Evaluation cases** | **Case number** | **Evaluation dimension** | **Evaluation approach** | **Evaluation indicators** | **Performance** | **Risks** |
| --- | --- | --- | --- | --- | --- | --- | --- | --- |
| Daniel Spitzl | May-25 | Simulated cases | 88 | Accuracy | Ground truth comparison | F1-score;  Mis-classification | F1 Score: 70%-84%;  Mis-classification proportion: 21.59%; | sensitive to prompts change |
| Kai Wang | May-25 | Real-world cases | 2056 | Accuracy | Ground truth comparison | AUROC;  AUPRC;  TPR;  FPR;  YI;  Brier Score | AUROC: 89.57%-92.13%;  AURPC: 88.21%-93.18%;  TPR: 75.72%-78.03%;  FPR: 0.00%-0.00%;  YI: 75.72-78.03%;  Brier Score: 8.05%-9.16%; | NI |
| Tuğgen Özcivelek | May-25 | Simulated questions | 35 | Accuracy;  Fairness | Expert rating;  Text assessment | Overall quality;  Accuracy;  Understandability;  Actionability;  Readability; | Overall quality: 5 (min: 4, max: 5, range: 1-5);  Accuracy: 5 (min:3, max: 5, range: 1-5);  Understandability: 87.8 (min: 63.6, max: 92.3, range: 0-100);  Actionability: 60 (min: 20, max: 70, range: 0-100);  FRES: 50.1 (min: 17.8, max: 71.6, range: 0-100);  FKGL: 9.4±1.8; | Inappropriate recommendation; misaligned content to patient needs and conditions |
| Mi Zhou | Jun-25 | Simulated questions | 3 | Accuracy;  Comprehensiveness;  Fairness | Researcher rating;  Text assessment | DISCERN;  Readability | V3:  DISCERN: 53.5-55 (range: 15-80);  FRES: 48.4-55.6 (range: 0-100);  FKGL: 8.3-10.1;  R1:  DISCERN: 55-56 (range 15-80);  FRES: 47.4-61.4 (range: 0-100);  FKGL: 7.2-9.0; | Inappropriate recommendation; misaligned content to patient needs and conditions |
| Junyang Ma | Jun-25 | Simulated cases | 1 | Accuracy;  Comprehensiveness | Expert rating | Decision rationality;  Strategic coherence;  Risk anticipation;  Reasoning transparency | Decision rationality: 3.75-4.25 (range: 1-5);  Strategic coherence: 3.6-4.2 (range: 1-5);  Risk Anticipation: 3.5-4.0 (range: 1-5);  Reasoning Transparency: 3.5-4.0 (range: 1-5); | Inappropriate recommendation; misaligned content to patient needs and conditions |

| **Author** | **Date** | **Evaluation cases** | **Case number** | **Evaluation dimension** | **Evaluation approach** | **Evaluation indicators** | **Performance** | **Risks** |
| --- | --- | --- | --- | --- | --- | --- | --- | --- |
| Olivier Niel | Jun-25 | Simulated cases | 10 | Accuracy | Expert rating | Overall accuracy;  Sub-domain accuracy | Overall accuracy: 76.2%;  Sub-domain accuracy: Diagnosis (100%);  Biological and imaging explorations (91.67%);  Drug and non-drug treatment (65.42%);  Logic and reasoning (61.9%) | Hallucination; inappropriate recommendation |
| Hilmi Anil Dincer | Jun-25 | Simulated questions | 20 | Accuracy;  Comprehensiveness;  Fairness | Expert rating;  Text assessment | Overall quality;  Comprehensiveness;  Readability | Overall quality: 92.5% appropriate; 7.5% incomplete responses;  Comprehensiveness: 95% (rated 5), 5% (rated 4);  FRES: 58.3±8.2 (range: 0-100);  FKGL: 8.7 (8.3-11.1);  CLK: 15.1 (14.2-16);  SMOG: 16.4±3.5; | NI |
| Xintong Wu | Jun-25 | Simulated cases | 48 | Accuracy;  Comprehensiveness | Ground truth comparison;  Expert rating | Accuracy;  Overall quality;  Completeness;  Clarity;  Usefulness | Accuracy:68%;  Overall quality: 5 (IQR: 4-5, range: 1-5);  Completeness: 4 (IQR: 4-5, range: 1-5);  Clarity: 5 (IQR: 4-5, range: 1-5);  Usefulness: 5 (IQR: 4-5, range: 1-5); | NI |
| Serhat Gurbuz | Jun-25 | Real-world questions | 5 | Accuracy;  Fairness | Expert rating | Overall quality;  Equity; | Overall quality: 7.4±1.2 (range: 1-10);  Equity: no significant difference across different age groups and genders | Inappropriate recommendation; misaligned content to patient needs and conditions |
| Onur Gültekin | Jun-25 | Simulated questions | 10 | Accuracy; Comprehensiveness;  Fairness | Expert rating;  Text assessment | Accuracy;  Clarity;  Completeness;  Consistency;  Readability | Accuracy: 3.9 ± 0.5 (range: 1-4);  Clarity: 3.9±0.3 (range: 1-4);  Completeness: 3.2±0.4 (range: 1-4);  Consistency: 4.0±0.0 (range: 1-4);  FRES: 61.3±6.0 (range: 0-100);  FKGL: 8.9±1.0 | NI |

| **Author** | **Date** | **Evaluation cases** | **Case number** | **Evaluation dimension** | **Evaluation approach** | **Evaluation indicators** | **Performance** | **Risks** |
| --- | --- | --- | --- | --- | --- | --- | --- | --- |
| Burcu Vural Camalan | Jun-25 | Simulated cases | 50 | Accuracy | Ground truth comparison; Expert rating | Accuracy | Diagnosis: 62%;  Treatment: 80% fully correct, 20% partially correct. | Inappropriate recommendation |

AUPRC = Area under the precision-recall curve; AUROC = Area under the receiver operating characteristic curve; CLI = Coleman-Liau Index; DISCERN = instrument for judging the quality of written consumer health information on treatment choices; FKGL = Flesch–Kincaid Grade Level; FPR = False positive rate; FRES = Flesch Reading Ease Score; NI = No information; SMOG = Simple Measure of Gobbledygook; TPR = True positive rate; YI = Youden index.

The table presents performance and risks extracted from studies assessing DeepSeek use in health care. Studies were presented in chronological order based on publication month.

**Figure S1. Evaluation of the bias risk in the included white literature using the JBI Checklist**

**
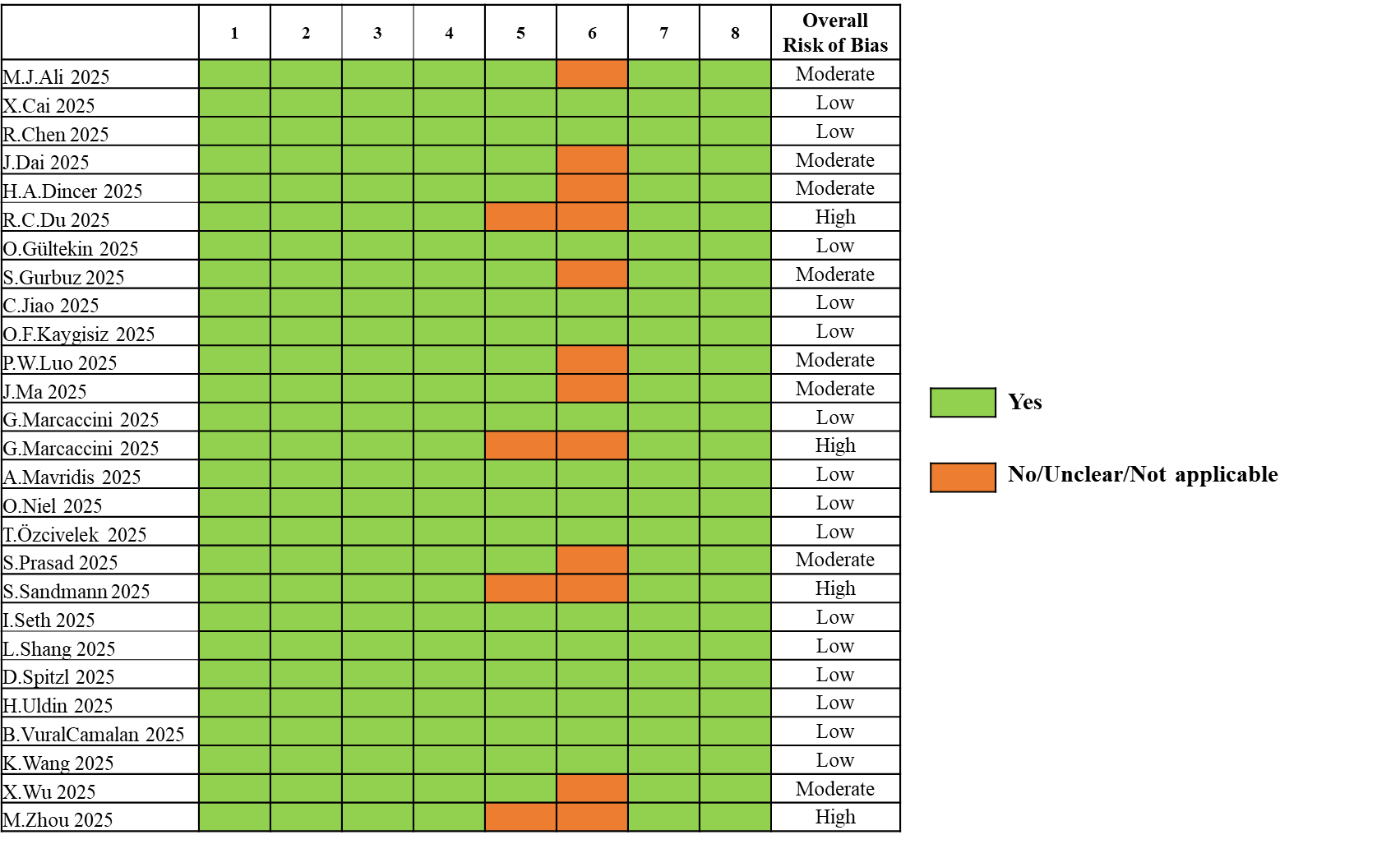
**

We utilized The Joanna Briggs Institute (JBI) Critical Appraisal Checklist for Analytical Cross Sectional Studies to assess the risk of bias. This checklist consists of eight questions, each scored as 1 point for "Yes" or 0 points for "No", "Unclear", or "Not Applicable". A study achieving a "Yes" response for all items receives a total score of 8. The risk of bias was categorized as follows: a score of 8 indicated low risk; a score of 7 indicated moderate risk; and a score of 6 or below indicated high risk. Any discrepancies between the two reviewers were resolved through discussion.

Among the included studies, four were rated as high risk[1-4], eight as moderate risk[5-12], and fifteen as low risk. [13-27]（figure 1）The most frequently identified weaknesses pertained to the identification of confounding factors (Question 5) and the statement of strategies to deal with them (Question 6).

1. Du RC, Zhu YC, Xiao YT, Yang BN, Lai YK, Zhou ZX, et al. Assessing the Capabilities of Novel Open-Source Artificial Intelligence-DeepSeek in Helicobacter pylori-Related Queries. Helicobacter. 2025 May-Jun;30(3):e70045. PMID: 40415185. doi: 10.1111/hel.70045.

2. Marcaccini G, Seth I, Xie Y, Susini P, Pozzi M, Cuomo R, et al. Breaking Bones, Breaking Barriers: ChatGPT, DeepSeek, and Gemini in Hand Fracture Management. J Clin Med. 2025 Mar 14;14(6). PMID: 40142791. doi: 10.3390/jcm14061983.

3. Sandmann S, Hegselmann S, Fujarski M, Bickmann L, Wild B, Eils R, et al. Benchmark evaluation of DeepSeek large language models in clinical decision-making. Nat Med. 2025 Aug;31(8):2546-9. PMID: 40267970. doi: 10.1038/s41591-025-03727-2.

4. Zhou M, Pan Y, Zhang Y, Song X, Zhou Y. Evaluating AI-generated patient education materials for spinal surgeries: Comparative analysis of readability and DISCERN quality across ChatGPT and deepseek models. International Journal of Medical Informatics. 2025 Jun;198. PMID: WOS:001450285000001. doi: 10.1016/j.ijmedinf.2025.105871.

5. Ali MJ. DeepSeek(TM) and lacrimal drainage disorders: hype or is it performing better than ChatGPT(TM)? Orbit. 2025 May 8:1-7. PMID: 40336348. doi: 10.1080/01676830.2025.2501656.

6. Dai J, Kim MY, Sutton RT, Mitchell JR, Goebel R, Baumgart DC. Comparative analysis of natural language processing methodologies for classifying computed tomography enterography reports in Crohn's disease patients. NPJ Digit Med. 2025 May 30;8(1):324. PMID: 40442294. doi: 10.1038/s41746-025-01729-5.

7. Dincer HA, Dogu D. Evaluating Artificial Intelligence in Patient Education: DeepSeek-V3 Versus ChatGPT-4o in Answering Common Questions on Laparoscopic Cholecystectomy. ANZ J Surg. 2025 Jun 11. PMID: 40495650. doi: 10.1111/ans.70198.

8. Gurbuz S, Bahar H, Yavuz U, Keskin A, Karslioglu B, Solak Y. Comparative Efficacy of ChatGPT and DeepSeek in Addressing Patient Queries on Gonarthrosis and Total Knee Arthroplasty. Arthroplast Today. 2025 Jun;33:101730. PMID: 40521295. doi: 10.1016/j.artd.2025.101730.

9. Luo PW, Liu JW, Xie X, Jiang JW, Huo XY, Chen ZL, et al. DeepSeek vs ChatGPT: a comparison study of their performance in answering prostate cancer radiotherapy questions in multiple languages. Am J Clin Exp Urol. 2025;13(2):176-85. PMID: 40400997. doi: 10.62347/uiap7979.

10. Ma J, Bu W, Wan M, Deng L, Cheng J. Comparative analysis of ChatGPT and DeepSeek in dynamic clinical decision-making: A progressive scenario-based evaluation. J Clin Anesth. 2025 Jun;104:111839. PMID: 40267548. doi: 10.1016/j.jclinane.2025.111839.

11. Prasad S, Langlie J, Pasick L, Chen R, Franzmann E. Evaluating advanced AI reasoning models: ChatGPT-4.0 and DeepSeek-R1 diagnostic performance in otolaryngology: a comparative analysis. American Journal of Otolaryngology. 2025 Jul-Aug;46(4). PMID: WOS:001491719100001. doi: 10.1016/j.amjoto.2025.104667.

12. Wu X, Huang Y, He Q. A large language model improves clinicians' diagnostic performance in complex critical illness cases. Critical Care. 2025 Jun 6;29(1). PMID: WOS:001504272000001. doi: 10.1186/s13054-025-05468-7.

13. Cai X, Geng Y, Du Y, Westerman B, Wang D, Ma C, et al. Utilizing Large language models to select literature for meta-analysis shows workload reduction while maintaining a similar recall level as manual curation. BMC Med Res Methodol. 2025 Apr 28;25(1):116. PMID: 40295957. doi: 10.1186/s12874-025-02569-3.

14. Chen R, Zhang S, Zheng Y, Yu Q, Wang C. Enhancing treatment decision-making for low back pain: a novel framework integrating large language models with retrieval-augmented generation technology. Front Med (Lausanne). 2025;12:1599241. PMID: 40438365. doi: 10.3389/fmed.2025.1599241.

15. Gültekin O, Inoue J, Yilmaz B, Cerci MH, Kilinc BE, Yilmaz H, et al. Evaluating DeepResearch and DeepThink in anterior cruciate ligament surgery patient education: ChatGPT-4o excels in comprehensiveness, DeepSeek R1 leads in clarity and readability of orthopaedic information. Knee Surg Sports Traumatol Arthrosc. 2025 Aug;33(8):3025-31. PMID: 40450565. doi: 10.1002/ksa.12711.

16. Jiao C, Rosas E, Asadigandomani H, Delsoz M, Madadi Y, Raja H, et al. Diagnostic Performance of Publicly Available Large Language Models in Corneal Diseases: A Comparison with Human Specialists. Diagnostics (Basel). 2025 May 13;15(10). PMID: 40428214. doi: 10.3390/diagnostics15101221.

17. Kaygisiz OF, Teke MT. Can deepseek and ChatGPT be used in the diagnosis of oral pathologies? Bmc Oral Health. 2025 Apr 25;25(1). PMID: WOS:001476777900008. doi: 10.1186/s12903-025-06034-x.

18. Marcaccini G, Seth I, Novo J, McClure V, Sacks B, Lim K, et al. Leveraging Artificial Intelligence for Personalized Rehabilitation Programs for Head and Neck Surgery Patients. Technologies. 2025;13(4):142. PMID: doi:10.3390/technologies13040142.

19. Mavridis A, Tegos S, Anastasiou C, Papoutsoglou M, Meditskos G. Large language models for intelligent RDF knowledge graph construction: results from medical ontology mapping. Front Artif Intell. 2025;8:1546179. PMID: 40352975. doi: 10.3389/frai.2025.1546179.

20. Niel O, Dookhun D, Caliment A. Performance evaluation of large language models in pediatric nephrology clinical decision support: a comprehensive assessment. Pediatr Nephrol. 2025 Oct;40(10):3211-8. PMID: 40461786. doi: 10.1007/s00467-025-06819-w.

21. Özcivelek T, Özcan B. Comparative evaluation of responses from DeepSeek-R1, ChatGPT-o1, ChatGPT-4, and dental GPT chatbots to patient inquiries about dental and maxillofacial prostheses. BMC Oral Health. 2025 May 31;25(1):871. PMID: 40450291. doi: 10.1186/s12903-025-06267-w.

22. Seth I, Marcaccini G, Lim K, Castrechini M, Cuomo R, Ng SK, et al. Management of Dupuytren's Disease: A Multi-Centric Comparative Analysis Between Experienced Hand Surgeons Versus Artificial Intelligence. Diagnostics (Basel). 2025 Feb 28;15(5). PMID: 40075834. doi: 10.3390/diagnostics15050587.

23. Shang L, Sha S, Hou Y. Evaluating Cardiovascular-Kidney-Metabolic Syndrome Knowledge in Large Language Models: A Comparative Study of ChatGPT, Gemini, and DeepSeek. Diabetes Technol Ther. 2025 May 27. PMID: 40421940. doi: 10.1089/dia.2025.0216.

24. Spitzl D, Mergen M, Bauer U, Jungmann F, Bressem KK, Busch F, et al. Leveraging large language models for accurate classification of liver lesions from MRI reports. Comput Struct Biotechnol J. 2025;27:2139-46. PMID: 40502931. doi: 10.1016/j.csbj.2025.05.019.

25. Uldin H, Saran S, Gandikota G, Iyengar KP, Vaishya R, Parmar Y, et al. A comparison of performance of DeepSeek-R1 model-generated responses to musculoskeletal radiology queries against ChatGPT-4 and ChatGPT-4o - A feasibility study. Clin Imaging. 2025 Jul;123:110506. PMID: 40381536. doi: 10.1016/j.clinimag.2025.110506.

26. Vural Camalan B, Doluoglu S, Taraf NH, Gunay MM, Ozlugedik S. ChatGPT versus DeepSeek in head and neck cancer staging and treatment planning: guideline-based study. Eur Arch Otorhinolaryngol. 2025 Jun 17. PMID: 40523995. doi: 10.1007/s00405-025-09524-4.

27. Wang K, Lin L, Zheng R, Nan S, Lu X, Duan H. Leveraging large language models for preoperative prevention of cardiopulmonary bypass-associated acute kidney injury. Ren Fail. 2025 Dec;47(1):2509786. PMID: 40442891. doi: 10.1080/0886022x.2025.2509786.

**Supplementary Data 1. An example case of how a hospital disclosure is mapped to the coding framework**

**Original materials (Translated materials)**

**Accessed at Sep 24, 2025 (**[**https://ch.shmu.edu.cn/main/news/content/id/492/pid/22955.html**](https://ch.shmu.edu.cn/main/news/content/id/492/pid/22955.html)**) Released at the official account of Fudan Pediatrics**

**DS-Dr. Xiaobu 2.0 is Here! Comprehensive Upgrade of Smart Healthcare at Fudan Pediatrics**

Release Date: March 10, 2025

Recently, the Children's Hospital of Fudan University have launched a newly upgraded "DS-Dr. Xiaobu 2.0" system, with localized deployment and application, further advancing the intelligent decision-making upgrade in pediatric healthcare.

**Basic Characteristics**

Deployment time;

AI name;

Deployment method

Dr. Xiaobu AI was independently developed by the Children's Hospital of Fudan University in collaboration with the School of Computer Science and the International Phenome Research Institute of Fudan University. Under the personal guidance and deployment of Academician Jin Li of Fudan University, and based on learning from over 9 million medical records, more than 100,000 inpatient cases, and covering over 7,000 pediatric diseases at the pediatric hospital, a cutting-edge intelligent diagnosis and treatment decision-making model was established, first launched in 2020.

**Medical specialty**

Through deep learning and data governance, the "DS-Dr. Xiaobu 2.0" version, which reflects the characteristics and quality of Fudan Pediatrics' clinical intelligent decision-making, deeply integrates the complex long-range reasoning capabilities of the DeepSeek large language model. It not only achieves intelligent upgrades across the entire medical service chain but also provides patients with more accurate personalized diagnosis and treatment plans, opening a new chapter in digital intelligent healthcare.

Patient Side: Seamlessly Integrating Professional Diagnosis with Daily Health Management Through Technology

By reshaping the children's medical experience with "technological warmth" and an innovative model of "precision diagnosis and treatment + emotional interaction," DS-Dr. Xiaobu 2.0 features a warm and cute cartoon image and caring dialogue. It is the first medical AI system in China to offer a full-process, humanistic service covering

**For** **patients: pre-consultation, during-consultation, and post-consultation**

**Pre-consultation:** DS-Dr. Xiaobu 2.0 quickly analyzes children's symptoms through natural language interaction technology. Parents can simply describe symptoms via text to receive accurate department recommendations and automatic appointment scheduling. It also thoughtfully provides notes for the hospital visit, navigation, and pre-consultation services, helping children and parents better understand the process.

**Targeted task and task type**

**During Consultation:** DS-Dr. Xiaobu 2.0 provides parents with detailed medication guidance, examination notes based on the latest evidence-based guidelines, diagnostic standards, and expert consensus, and pushes wait-time reminders and in-hospital navigation services.

**Targeted task and task type**

**Post-consultation:** For the health management of children with chronic diseases, DS-Dr. Xiaobu 2.0 forms a closed-loop service by pushing medication guidance, follow-up suggestions, and health management services, combined with re-examination reminders, practicing a "treatment-management-prevention" closed-loop service model. This achieves seamless integration from professional diagnosis to daily health management, offering 24 hours warm care.

**Targeted task and task type**

**For clinicians: Intelligent Decision-Making Frees Doctors to Focus on Clinical Productivity**

The DS-Dr. Xiaobu 2.0 system, through deep integration with DeepSeek, enhances diagnostic accuracy, personalized support, and high-risk warning capabilities, promoting the development of intelligent and personalized medical services. Simultaneously, through intelligent medical record management and treatment data quality control, it significantly improves diagnostic and treatment efficiency, truly allowing doctors to focus on "key decisions" and freeing up clinical productivity.

Through deep integration with the DeepSeek large language model, the reasoning ability of DS-Dr. Xiaobu 2.0 is fully upgraded. Especially when handling complex conditions and co-morbidities, it can provide more precise diagnostic support, with diagnostic consistency exceeding 95%. This greatly reduces the risk of misdiagnosis and missed diagnosis while ensuring diagnostic consistency.

**Targeted task and task type**

Leveraging the pediatric retrieval-augmented knowledge base built by Fudan Pediatrics Hospital, DS-Dr. Xiaobu 2.0 uses big data analysis to provide doctors with real-time, comprehensive decision support, assisting in personalized diagnosis. This includes accurately identifying high-risk conditions for early warning, predicting potential disease progression paths in real-time, providing severity analysis to help doctors identify potential complications early, and suggesting timely adjustments to treatment plans to optimize efficacy and improve outcomes.

**For administrative managers: Enabling Higher Quality Modeling and More Precise Diagnosis/Treatment**

The improvement of overall hospital efficiency by DS-Dr. Xiaobu 2.0 is also reflected in scientific research innovation, medical supply management, and resource scheduling. Through a dynamic medical knowledge base, the system provides real-time data support for researchers, uncovering new trends in medical innovation. Deeply integrated with the hospital's Supply Processing and Distribution (SPD) system, it enables intelligent management of medical supplies. An intelligent medical resource scheduling system has been established, successfully creating the "Dr. Xiaobu Decision Companion" to help hospitals monitor operational status in real-time and optimize resource allocation. For example, the system can predict future patient flow, improving bed turnover rates and resource utilization efficiency based on factors like bed occupancy and surgery schedules.

**Targeted task and task type**

In 2022, the hospital developed a Knowledge, Attitude, and Practice (KAP) scale for clinical AI application. Through a sample survey of 332 medical staff, ethical governance issues were systematically reviewed. By establishing a "1-3-5" evaluation index system (1 application scenario - 3 stakeholder perspectives - 5 evaluation dimensions), the AI-driven clinical decision support system undergoes full-process dynamic monitoring. This ensures the technology operates transparently, fairly, and responsibly during implementation, while continuously optimizing and enhancing trust among medical staff and patients. Furthermore, the DeepSeek large model is deployed locally and privately within the pediatric hospital's environment, strictly ensuring data security.

**LLMs evaluation**

Professor Wang Yi, President of the Children's Hospital of Fudan University, stated that the launch of DS-Dr. Xiaobu 2.0 marks a comprehensive breakthrough for domestically developed large language models in the field of pediatric healthcare. From initial data alignment and modeling to continuous iteration, from focusing on data governance and building large hospital decision-making models, to integrating global knowledge bases for combined internal and external data support, the decision support functionality has been refined. Now, with the enhancement of DeepSeek, higher quality modeling and more precise diagnosis and treatment are achieved. The pediatric hospital is also considering building a "Dr. Xiaobu Giant," tailor-made expert digital twins to achieve more optimized and intelligent consultations.

**Mapping process**

This case study uses the news article **“DS-Dr. Xiaobu 2.0 is Here! Comprehensive Upgrade of Smart Healthcare at Fudan Pediatrics”** as an example to demonstrate step-by-step how information is extracted from the news and mapped to the coding framework.

**Step 1: Basic Information Extraction**

Information extraction is based on the original news text, while coding involves mapping the information to the categories of the coding framework. Information not explicitly mentioned is marked as “Not mentioned”.

Key information is extracted from the news article. The extraction is based directly on the original text or infers obvious meanings (e.g., the hospital’s province). The results are as follows:

**Step 2: Coding process**

For information requiring coding, relevant details are identified within the news article (grey area marked in the original file) and then mapped to the respective categories of the coding framework. Items not mentioned are marked “Not mentioned”; items mentioned are recorded with their specific content. The coding results are as follows:

- **Healthcare Institution Information:**

**Name:** The Children's Hospital of Fudan University

**Tier/Grade**: Tier 3, Grade A

**City**: Shanghai

**Ranking**: Fudan Ranking A+++

- **Large Language Model Basic Information:**

**Name:** DS-Dr. Xiaobu 2.0

**Version:** Not mentioned

**News Date:** 2025/03/10 (Publication date)

**Deployment Method**: Local deployment (The article explicitly mentions “conducting local deployment and application”)

**Deployment Department:** Target Department: Pediatrics (The system is for a pediatric hospital and “pediatrics” is mentioned multiple times)

- **Targeted Tasks (Code to the most specific task level possible):**

**a) Clinical Diagnosis & Decision Support**

i. Diagnosis Recommendation/diagnosis formulation: Yes (Mentions “diagnostic support”, “precise diagnosis”)

ii. Treatment Plan Recommendation: Yes (Mentions “treatment plan”, “optimizing treatment efficacy”)

iii. Medication Recommendation: Yes (Mentions “medication guidance”)

iv. Clinical note Material Processing (Composition, Summarization, Generation, etc.): Not mentioned

v. Hospital Management / Operational Decision Support: Yes (Mentions “hospital management decisions”, “resource scheduling”)

**b) Patient Management & Services**

i. Patient Education: Not mentioned

ii. Treatment Adherence Support: Not mentioned

iii. Health Monitoring: Not mentioned

iv. Appointment Coordination / Triage: Yes (Mentions “appointment registration”, “triage”)

**c) Research & Education**

i. Literature Synthesis: Not mentioned

ii. Clinical Knowledge Assessment: Not mentioned

iii. Medical Education: Not mentioned

iv. Research Protocol Support: Not mentioned

- **Evaluation Approach**

a) Not Evaluated / No Mention in sources: Not mentioned

b) Internal Review (No publicly available evaluation results): Not mentioned

c) Internal Review (Self-published evaluation results): Yes (Article explicitly mentions “diagnostic consistency exceeding 95%”)

d) External Review (Publicly published testing articles): Not mentioned

- **Evaluation Dimensions**

a) Accuracy (Measures how close the LLM output is to the true or expected answer): Yes (Mentions "diagnostic consistency")

b) Comprehensiveness (Measures how well an LLM’s output coherently and concisely addresses all aspects of the task and reference provided.): Not mentioned

c) Factuality / Hallucination (Measures how an LLM’s output for a specific task originates from a verifiable and citable source. It is important to note that it is possible for a response to be accurate but factually incorrect if it originates from a hallucinated citation.): Not mentioned

d) Robustness (Measures the LLM’s resilience against adversarial attacks and perturbations such as typos.): Not mentioned

e) Fairness, bias, and toxicity (Measures whether an LLM’s output is equitable, impartial, and free from harmful stereotypes or biases, ensuring it does not perpetuate injustice or toxicity across diverse groups.): Not mentioned

f) Deployment metrics (Measures the technical and parametric details of an LLM to generate a desired output.): Not mentioned

g) Calibration and uncertainty (Measures how uncertain or underconfident an LLM is about its output for a specific task.): Not mentioned

- **Potential Risks & Mitigation Strategies**

**a) Clinical-Related**

i. Hallucination of nonexistent symptoms/diseases & Mitigation: Not mentioned

ii. Inappropriate treatment recommendations & Mitigation: Not mentioned

iii. Inadequate handling of medical uncertainty & Mitigation: Not mentioned

iv. Clinician Over-reliance & Mitigation: Not mentioned

**b) Patient-Related**

i. Misalignment between AI recommendations and patient conditions & Mitigation: Not mentioned

ii. Inconsistent quality of patient materials & Mitigation: Not mentioned

iii. Inconsistent patient education quality or inappropriate self-management advice & Mitigation: Not mentioned

iv. Miscommunication of risks/benefits & Mitigation: Not mentioned

**c) Teaching & Research-Related:**

i. Potential reinforcement of outdated medical knowledge & Mitigation: Not mentioned

ii. Risk of fabricated references or studies & Mitigation: Not mentioned

iii. Uncritical adoption by medical trainees & Mitigation: Not mentioned

- Management Measures

a) Establish Specific Task Evaluation Plans (Clear clinically relevant metrics, not just technical standards): Yes (Mentions “1 application scenario - 3 stakeholder perspectives - 5 evaluation dimensions”)

b) Adopt Multi-stage Model Performance Validation Processes (Pre-implementation, Ongoing monitoring during implementation): Yes (Mentions “dynamic monitoring”)

c) Develop Integrated Training & Educational Materials for Patient and Clinician AI Use: Not mentioned

d) Establish Stable Governance Structure (Including data security, privacy oversight plans, regular potential risk assessments): Not mentioned
